# Combined phage therapy and faecal microbiota transplantation to treat recurrent urinary tract infection: a case series

**DOI:** 10.1038/s41564-026-02409-0

**Published:** 2026-07-30

**Authors:** Shawna McCallin, Annika Y. Classen, Simone C. Lieberknecht-Jouy, Fatih Abdula, Sarah Dugas, Oliver Gross, Hendrik Koliwer-Brandl, Swenja Lassen, Jens Scheidegger, Matthew Dunne, Andre Kahles, Jonas Eigler, Fedja Farowski, Paul G. Higgins, Sonja Milek, Oksana Chemych, Jiemin Du, Martin J. Loessner, Thomas M. Kessler, Maria J. G. T. Vehreschild, Lorenz Leitner, Lena M. Biehl

**Affiliations:** 1 Department of Neuro-Urology, Balgrist University Hospital, University of Zurich, Zurich, Switzerland; 2 Spinal Cord Injury Center, Balgrist University Hospital, University of Zurich, Zurich, Switzerland; 3 The LOOP Zurich, Medical Research Center, Zurich, Switzerland; 4 ESGNTA - ESCMID Study Group for Non-Traditional Antibacterials, Basel, Switzerland; 5 ESGUTI - ESCMID Study Group for Urinary Tract Infection, Basel, Switzerland; 6 University of Cologne, Faculty of Medicine, and University Hospital Cologne, Department I of Internal Medicine, Cologne, Germany; 7 German Center for Infection Research (DZIF), Partner Site Bonn/Cologne, Cologne, Germany; 8 University of Cologne, Faculty of Medicine, Institute for Medical Microbiology, Immunology and Hygiene, and University Hospital Cologne, Cologne, Germany; 9 Department of Internal Medicine, Infectious Diseases, Goethe University Frankfurt, University Hospital Frankfurt, Frankfurt am Main, Germany; 10 Institute of Medical Microbiology, University of Zurich, Zurich, Switzerland; 11 Institute of Food Nutrition and Health, ETH Zurich, Zurich, Switzerland; 12 Biomedical Informatics Group, Department of Computer Science, ETH Zurich, Zurich, Switzerland; 13 Swiss Institute of Bioinformatics, Lausanne, Switzerland; 14 Medical Informatics, University Hospital Zurich, Zurich, Switzerland; 15 Department of Infectious Diseases with Epidemiology, Academic and Research Medical Institute, Sumy State University, Sumy, Ukraine; 16 Fraunhofer Institute for Translational Medicine and Pharmacology ITMP, Frankfurt am Main, Germany; 17 Fraunhofer Cluster of Excellence Immune-Mediated Diseases CIMD, Frankfurt am Main, Germany; 18 ESGHAMI - ESCMID Study Group for Host and Microbiota Interactions, Basel, Switzerland

**Keywords:** Bacterial infection, Bacteriophages

## Abstract

Recurrent urinary tract infections are recalcitrant and difficult-to-treat bacterial infections that primarily affect women. Here we combine phage therapy with faecal microbiota transplantation to decolonize the urinary and intestinal reservoirs of patients with recurrent urinary tract infections. Three women received oral and intravesical phage therapy for 8 days, and two underwent subsequent faecal microbiota transplantation. Treatments were well tolerated, and although *Escherichia coli* was detected in follow-up samples, patients experienced none or fewer and less severe episodes of urinary tract infection 24 months post-treatment.

## Abstract (web summary)

In a small case series, combined phage therapy and faecal microbiota transplantation was safe and led to sustained reductions in recurrence, severity and antibiotic use in women with recurrent urinary tract infections, even without complete eradication of *Escherichia coli*.

## Main

Recurrent urinary tract infection (rUTI) is a difficult-to-treat condition defined as two urinary tract infections (UTIs) within 6 months or three UTIs within the past year^1^. rUTIs are a leading cause of outpatient antibiotic use, accounting for >15% of all prescriptions^2–4^. This suggests that current treatment options, which predominantly rely on antibiotics, have limited success. Alternative therapeutic strategies such as phage therapy (PT) to remove pathogens and faecal microbiota transplantation (FMT) to restore microbiota diversity and prevent further recurrence have been proposed to address this clinical challenge, which have, until now, not been used together^5–7^. Here we report our rationale and first experience of PT combined with FMT for female patients with rUTI colonized by a stable clone of *Escherichia coli*^8^.

Three female patients, between 20 and 70 years of age, presented with long-standing histories of rUTI (5–44 years), requiring 4–10 antibiotic treatments within 12 months preceding experimental intervention and resulting in substantial impairment of daily functioning. All patients had microbiologically confirmed *E. coli* infections and reported partial or no response to an extensive array of conventional therapies, leading to a substantial decrease in quality of life and frustration over the lack of sustainable therapeutic options (Supplementary Information 1).

**Extended Data Fig. 1:**
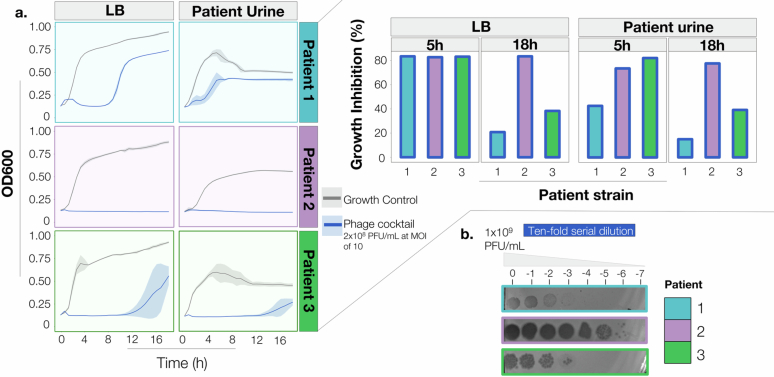
Phage susceptibility testing. **a**, Turbidity reduction assays (TRAs) measuring the effect of the phage cocktail on bacterial growth of pre-treatment patient isolates in Luria broth (LB) and in patient urine. Growth was measured by optical density at 600 nm (OD_600_) over time and is represented as growth curves and as percent growth inhibition in bar charts at 5 h and 18 h. Grey lines indicate growth controls and blue lines indicate phage cocktail treatment at 2 × 10^8^ PFU/mL, corresponding to an MOI of 10. Shaded areas indicate variation between technical replicate wells, n = 3. Percent inhibition (PI) was calculated from the area under the curve (AUC) relative to the corresponding growth control and is shown as a single AUC-derived value for each patient isolate, medium, and time point. The TRA experiment was performed once for each patient isolate and medium condition in technical triplicates. **b**, Spot assays using ten-fold serial dilutions, starting from 1 × 10^9^ PFU/mL in LB medium. The spot assay image shown was derived from a single experimental per patient (n = 3) isolate. Source data

**Extended Data Fig. 2:**
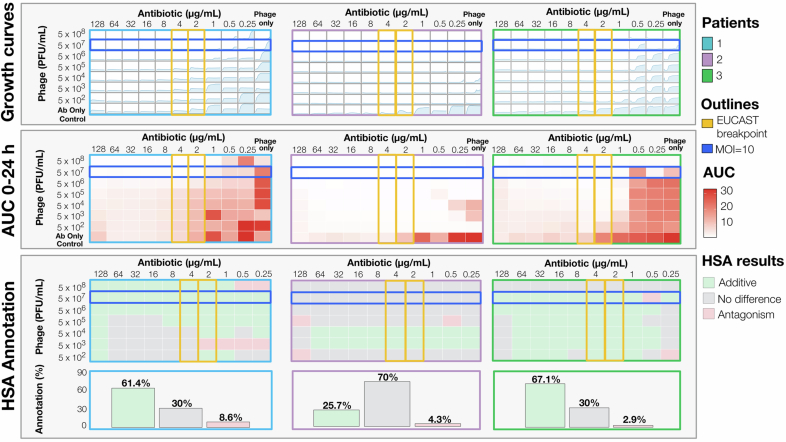
Phage-antibiotic combination testing. Bacterial growth of patient isolates was measured across a two-dimensional matrix of phage and antibiotic concentrations, including phage-only and antibiotic-only control conditions. Phage concentrations ranged from 5 × 10^2^ to 5 × 10^8^ PFU/mL and antibiotic concentrations ranged from 0.25 to 128 µg/mL. Each phage-antibiotic condition was tested once. Top, Growth curves for each condition. Middle, Heatmaps of area under the curve (AUC) over 0-24 h for each phage-antibiotic combination. The colour scale indicates AUC values. Yellow outlines mark the EUCAST breakpoint concentration(s), and blue outlines mark the MOI = 10 condition. Bottom, Interaction outcomes annotated using the highest single agent (HSA) model based on AUC and classified as additive, no difference, or antagonism. Bar plots summarize HSA outcomes per patient isolate. Percentages were calculated from n = 70 single-measurement phage-antibiotic combination conditions per patient isolate. Source data

As part of the patient evaluations for PT, bacterial strains were collected from urine samples over 6 months. Pre-treatment *E. coli* isolates were found to be consistently susceptible to co-trimoxazole (Supplementary Table 1) and to an existing two-phage cocktail in solid and liquid media and patient urine (Extended Data Fig. 1). The two-phage cocktail consisted of two wild-type *E. coli* phages, E2 (accession OL870316) and phi41S, belonging to the genera *Tequatrovirus* and *Phapecoctavirus*, respectively, which have complementary host ranges and use different receptors^9^. Both phages were isolated from environmental wastewater sources in Switzerland and were purified for these treatments using caesium chloride density gradient ultracentrifugation. Phage preparations underwent external quality control before use (Supplementary Information 2 and 3). Phage–antibiotic interaction testing demonstrated an additive or neutral effect between the treatments (Extended Data Fig. 2).

**Fig. 1:**
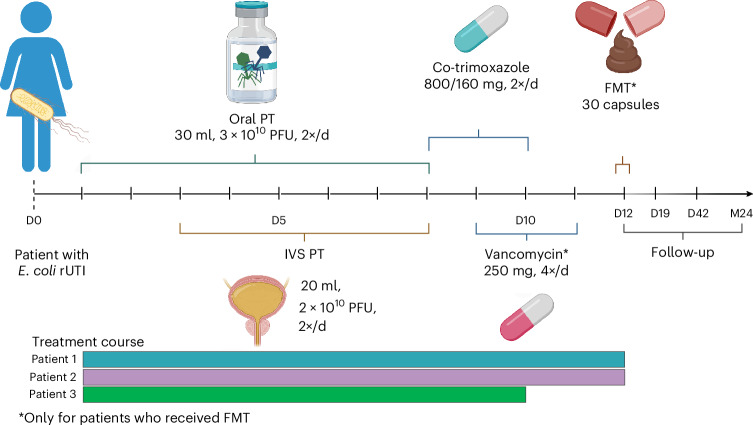
Treatment overview. Treatment overview of PT, antibiotics and FMT. Oral PT was given twice daily from day 1 to day 8, and IVS PT was added from day 3 to day 8. Co-trimoxazole was given from day 8 to day 10. Vancomycin pre-treatment and FMT were performed only in patients who received FMT, as indicated in the figure (*). Coloured horizontal bars indicate the treatment course for each patient. Microbiological follow-up was continued for 30 days (D) after FMT, and clinical follow-up was continued for 24 months (M). Figure created in BioRender; Leitner, L. https://biorender.com/10lcg50 (2026).

The patients were scheduled for PT outside of acute episodes, and the phage cocktail was applied orally twice daily for 2 days before adding intravesical (IVS) administration via intermittent catheterization twice daily for an additional 6 days (Fig. 1). All patients received co-trimoxazole orally, and two patients opted to receive FMT via orally administered capsules. Follow-up samples were collected until day 30 after FMT, and clinical follow-up was continued for 24 months. Additional information about the products and dosing regimen is available in the Methods.

**Extended Data Fig. 3:**
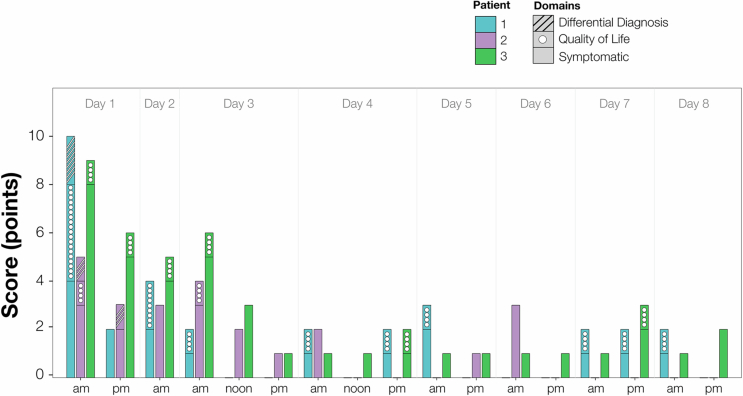
Acute Cystitis Symptom Scores. Individual patient values of the Acute Cystitis Symptom Score (ACSS) before administration and at each study visit, including sum scores for the symptomatic, quality-of-life, and differential-diagnosis domains. Domain patterns are defined in the figure key. Data are shown for n = 3 patients, with the color indicating the different patients. Each bar represents one patient-level ACSS assessment at the indicated time point. The Patient 1, Day 8, pm ACSS assessment was not recorded (∞).

**Extended Data Fig. 4:**
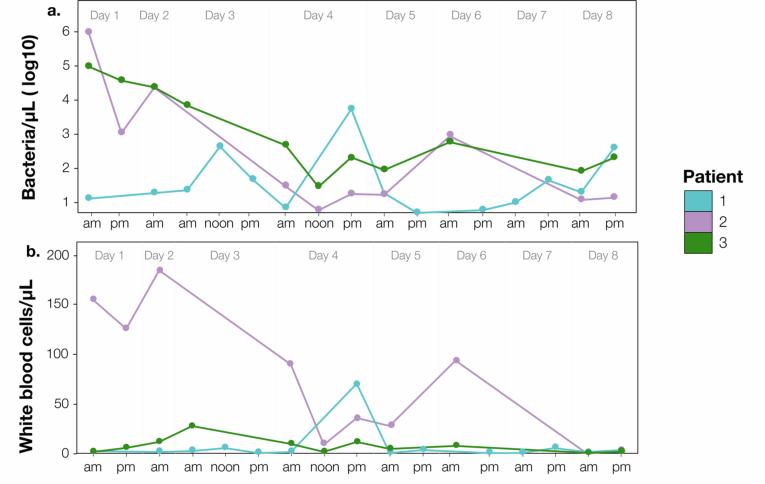
Urinalysis parameters. **a**, Total bacterial counts in urine per µL measured by flow cytometry using the Sysmex UF-5000 before administration and at each study visit, displayed on a log_10_ scale. These bacterial counts are not specific to *E. coli*. For Patient 2, the Day 1, am bacterial count exceeded the upper limit of quantification and was plotted at the highest reportable value. **b**, Total white blood cell counts in urine per µL measured by flow cytometry before administration and at each study visit. Line colours indicate individual patients. Data are shown for n = 3 patients. Each point represents one urine measurement from one patient at the indicated time point.

**Extended Data Fig. 5:**
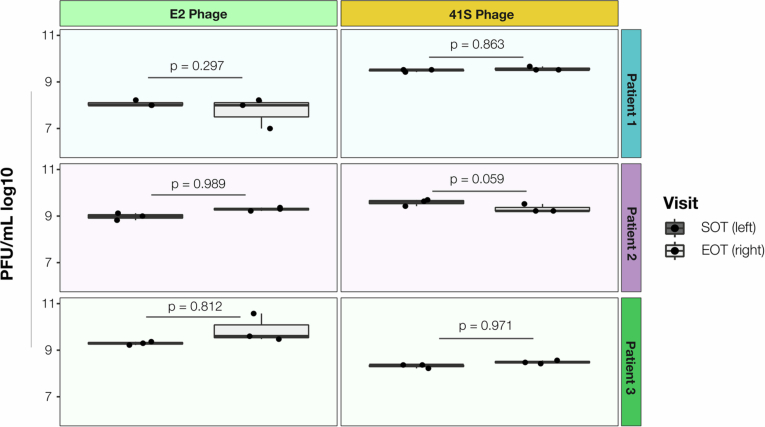
Serum neutralization of phages at start and end of therapy. Serum neutralization of phages (E2, left column; 41S, right column) at start of treatment (SOT) versus end of treatment (EOT). Residual phage titers after exposure to patient serum were measured by spot assay and are shown as log_10_ PFU/mL per patient and per phage. Points indicate technical replicates, with n = 3 technical replicates per patient, phage, and visit. Boxes show medians and interquartile ranges. One-sided Welch’s t-tests were used to compare residual phage titers at EOT versus SOT. Exact p-values are shown in the figure. Source data

The treatments were well tolerated, and no adverse effects were observed. Kidney and liver function parameters remained stable. Although patients were treated outside of acute episodes, they presented with persistent low-grade (oligosymptomatic) symptoms. PT was associated with a reduction in these chronic low-grade symptoms, demonstrated by improvement in symptoms (average reduction, 5 to 1) and quality of life (average reduction, 2 to 0) domains of the acute cystitis symptom score (Extended Data Fig. 3). Leukocyte counts determined by urine flow cytometry decreased during phage application, whereas bacterial counts, which were non-specific to *E. coli*, fluctuated (Extended Data Fig. 4). No change in serum neutralization to the phage cocktail was observed over the duration of PT (Extended Data Fig. 5).

**Fig. 2:**
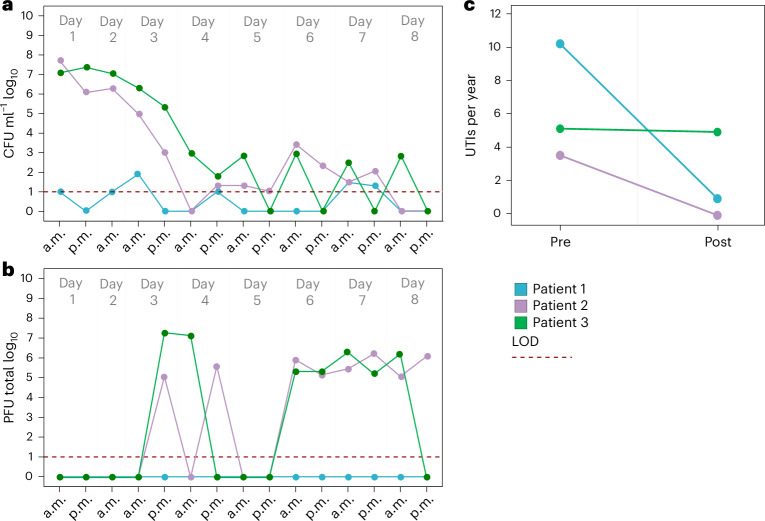
Clinical and microbiological outcomes. **a**, *E. coli* titres in urine measured pre-administration at each visit as colony-forming units per millilitre (CFU ml^−^^1^, log_10_). Each point represents one urine sample from one patient at the indicated time point. **b**, Total amount of phage in urine measured pre-administration at each visit as plaque-forming units (PFU total, log_10_). Each point represents one urine sample from one patient at the indicated time point. **c**, Frequency of UTIs, shown as episodes per year before treatment and during 24 months after treatment. Each point represents one patient-level clinical value for the indicated period. Colours indicate individual patients. The dashed red line indicates the limit of detection (LOD). a.m. indicates morning visits and p.m indicate afternoon/evening visits. Data are shown for *n* = 3 patients. Source data

Two patients presented with high *E. coli* titres in the urine at the start of treatment. Pathogen titres decreased substantially at the onset of IVS administration, reducing *E. coli* by 3 log_10_ within 2 h of the first phage IVS installation. *E. coli* titres remained low or undetectable over the treatment period, and a 7-log_10_ decrease in urine *E. coli* was achieved for two patients (Fig. 2a). However, prolonged and durable eradication was not achieved, as *E. coli* was detected in subsequent follow-up samples (Extended Data Table 1).

**Extended Data Fig. 6:**
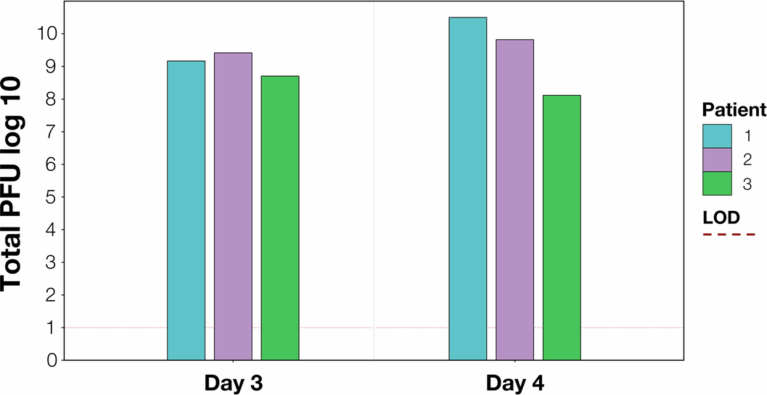
Viable phage recovery in urine at first void. A dose of 2 × 10^10^ PFU was applied intravesically, and the phage cocktail was retained in the bladder until the next void. Phage concentrations in urine were quantified using dilution spot assays (PFU/mL) and multiplied by the total volume of voided urine. Total recovered phage is shown as PFU on a log_10_ scale. Colours indicate individual patients. The dashed red line indicates the limit of detection (LOD). Data are shown for first-void urine samples collected per patient on day 3 and day 4. This recovery experiment was performed once, with one measurement per first-void urine sample. Source data

Phage was quantified in the urine before each administration (Fig. 2b). At two visits, first-voided urine after phage IVS instillation was collected (Extended Data Fig. 6). Phage was washed out between administrations for patient 1 with low/undetectable *E. coli* titres, indicating host-dependent pharmacokinetics. For the other two patients, phage reached a maximum of 1.6 × 10^6^ and 1.33 × 10^7^ PFU ml^−^^1^ in urine, and the presence of phage between visits was maintained 4 days after the start of treatment (average 5.8 × 10^5^ PFU ml^−^^1^). No phage was detected in the urine at the first follow-up, 4 days after PT.

**Extended Data Fig. 7:**
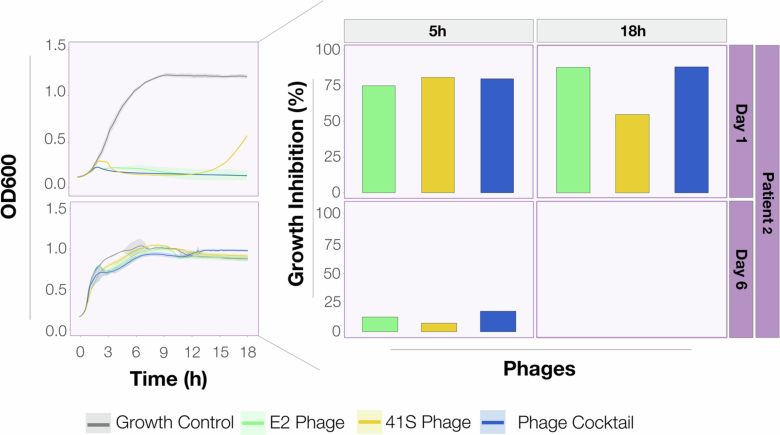
Phage susceptibility testing of Patient 2 isolates during phage treatment. Susceptibility of *E. coli* isolates from Patient 2 urine at day 1 and day 6, before antibiotic exposure, to the individual phages and the phage cocktail used for treatment. Growth curves show OD_600_ over time for growth control, E2 phage, 41S phage and phage cocktail conditions. Percent inhibition was calculated from the area under the OD_600_ curve relative to the corresponding growth control and is shown as a single AUC-derived value for each isolate, phage condition, and time point at 5 h and 18 h. The TRA experiment was performed once for each isolate and phage condition in technical triplicates. Source data

**Extended Data Fig. 8:**
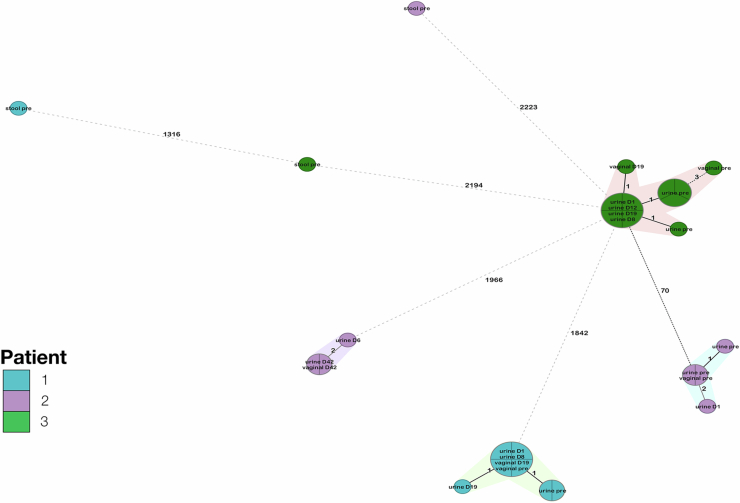
Minimum-spanning tree of patient *E.coli* isolates. *E. coli* isolates from urine, vaginal swabs, or stool were whole genome sequenced and analysed. Isolates are labelled by the source (stool, urine or vaginal) and coloured by patient. Line thickness represents the degree of relatedness. Numbers between the nodes show the numbers of alleles that differ between the strains. Shaded areas show clonality within the threshold of 10 alleles. Image generated using MBioSEQ Ridom Typer based on 2513 target genes.

In terms of phage resistance, phage susceptibility was similar in all isolates collected from patients 1 and 3 over the course of treatment, whereas a change was detected in the isolates from patient 2 (day 5/6) (Extended Data Fig. 7). Whole-genome sequencing showed that this decrease in phage susceptibility was not due to the development of phage resistance, but due to a phylogenetically distinct strain (Extended Data Fig. 8). This replacement strain remained stable in subsequent patient samples and, unlike the initial strain, was sensitive to ciprofloxacin (Supplementary Table 1). The urine and vaginal *E. coli* titres of patients 1 and 3 were observed to be stable via whole-genome sequencing throughout treatment; however, the replacement strain of patient 2 differed in 2,076/2,513 target alleles, lacked the determinants for fluoroquinolone resistance (phenotypically confirmed) and *fimB*, and instead carried an operon for F1C fimbriae (of lower virulence than type 1 fimbriae in the initial strain) and an iron acquisition gene cluster (Supplementary Table 2). This finding underscores the importance of strain-level microbiological evaluation of PT effects, as strain substitution may occur.

**Extended Data Fig. 9:**
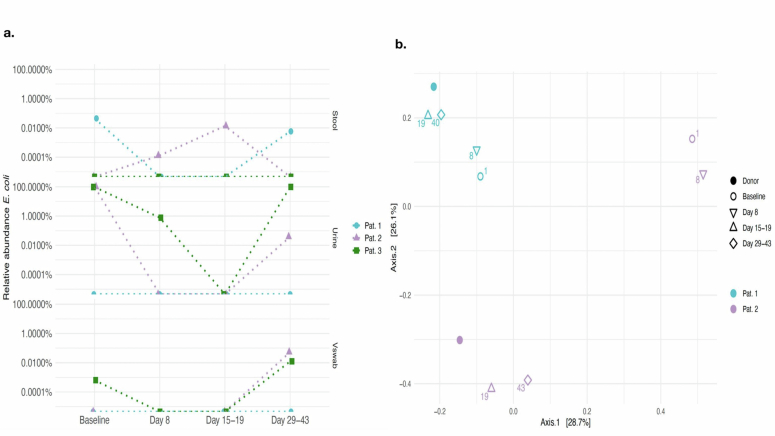
Shotgun metagenomic sequencing of patient samples. **a**, Relative abundance of *E. coli* in the different body materials over time. **b**, Principal coordinate (PCoA) analysis illustrating Bray-curtis beta diversity of stool samples. A shift towards the donor microbiota composition can be seen in the samples following FMT. Small numbers next to the symbols indicate day of sampling. Timing of samples: Baseline, Prior any treatment; Day 8, After phage treatment but before FMT; Day 15-19, 7 days after FMT; Day 29-43, 28-30 days after FMT in Patient (Pat.) 1 and 2.

Using shotgun metagenomic sequencing, we observed variable *E. coli* abundances in urine, vaginal and stool samples (Extended Data Fig. 9a) and negligible or very low levels of *E. coli* in the stool metagenomes of FMT donors (<0.000001% of reads). Beta-diversity analysis of the two FMT recipients confirmed a shift in the intestinal microbiota towards donor composition indicative of engraftment (Extended Data Fig. 9b).

The clinical effect of our treatment approach was assessed by the frequency of UTI episodes after treatment (Fig. 2c). Patient 1 experienced a decrease in UTI episodes, self-reported a decrease in symptom severity and required no further antibiotic treatments. Patient 2 had no further episodes during the 24-month follow-up. Patient 3, who did not undergo FMT, self-reported a decrease in symptom severity and the frequency of antibiotic treatment, but no decrease in the frequency of symptom occurrence. Patient perspectives are provided in Supplementary Information 4.

These findings are limited by the small case number and biases inherent to case reports, including the lack of comparators and blinding, and our reporting is in line with the CARE guidelines for case series (Supplementary Information 5). There is a general lack of evidence from randomized controlled trials (RCTs) of PT for UTI, with the only completed phase II RCT study for UTI showing phage to be non-inferior to antibiotics but non-superior to placebo^10^. A phase II clinical trial combining intravenous and IVS PT with orally administered antibiotics is ongoing (NCT05488340)^11^. Although there is extensive evidence and guidelines for the use of FMT in recurrent *Clostridioides difficile* infections, FMT is also being studied for the eradication of multidrug-resistant organisms and—so far, only in case series—for secondary prevention in rUTI^12–14^.

Despite its limitations, the experience and observations of using PT + FMT presented here highlight important considerations for future evaluation of these therapies in an RCT setting. One ongoing debate in UTI trials, as well as those for PT in general, is the use of microbiological endpoints^15,16^. Our observations indicate that bacterial concentrations varied throughout the treatment, often below the threshold for infection criteria; however, sustained eradication with phage alone may not be achievable or even necessary for a clinical effect. Even after co-trimoxazole treatment, *E. coli* was detected in clinical samples within a 30-day follow-up in all patients. For patient 2, the eradication of the initial strain was achieved, followed by the emergence of a different strain, one that has not caused an infection in the patient since treatment. From a clinical perspective, the patients experienced sustained benefits, including reduced antibiotic use, fewer or no recurrences, and improved quality of life. Although preliminary and obtained outside of an RCT, these findings suggest that clinical endpoints may be separate from microbiological measures and that the former should take precedence.

We found that oral administration alone did not result in phage detection in the urine, but that after several days of IVS instillation, phage titres were maintained between administrations. These findings should be further explored in RCTs to determine what factors support sustained phage titres in vivo (for example, loading dose, time, dose frequency and dose concentration). Treatment protocols remain highly variable between case reports, and in efforts to support standardization, the results of these cases have been documented in the International Phagistry, the anonymous registry for patients treated with PT^17^.

Given the high prevalence of UTIs in clinical practice and the consequently elevated rates of antibiotic use, the value of PT for UTI treatment should be further explored as a high-impact indication. In addition, other microbiome-based therapies, such as FMT, could be a useful combination treatment strategy to provide long-term modifications of microbial reservoirs. FMT was used here to reduce the likelihood of recurrences of UTI owing to a more protective microbiome, something that PT alone cannot achieve. The clinical utility of any new treatment option should be duly tested in a well-controlled RCT that sufficiently investigates both clinical and microbiological changes to gain a deeper understanding of the mechanisms of action that support sustainable relief from rUTI.

## Methods

### Patients

All three patients were seen in the rUTI clinics of Frankfurt or Cologne University Hospital, Germany. The decision to offer this experimental treatment was based on the fact that *E. coli* was persistently detected in those patients and that they had unsuccessfully undergone a wide variety of treatment and prophylaxis approaches for rUTI. The patients received PT at the Balgrist University Hospital, Zurich, Switzerland, and FMT at Cologne University Hospital, Cologne, Germany. Experimental treatments were provided free of cost, and the patients did not receive any compensation.

The patients gave written informed consent for individual treatment attempts and for the collection of biosamples for related research by agreeing to the biobanking protocol for Improving the Diagnosis of Severe Infections in Immunocompromised Patients (Ethics Committee of Cologne University Hospital, study ID 08-160). For the phage treatments, the patients gave written informed consent for an experimental therapy in the individual setting and general consent for the reuse of data and material.

The cases presented here are reported in line with the CARE guidelines for case reports (Supplementary Information 5) and in compliance with the principles of the Declaration of Helsinki.

### Dose regimen and treatment details

The dosage of the phage administrations was decided based on the literature and feasibility. The phage cocktail was applied orally twice daily (30 ml; 3 × 10^10^ PFU per dose), 30 min after gastric neutralization with 1 g sodium bicarbonate powder dissolved in 50 ml of water for 2 days (D1 and D2) before adding IVS phage administration via intermittent (in-and-out) catheterization using standard hydrophilic-coated single-use catheters twice daily (20 ml; 2 × 10^10^ PFU per dose) in the morning and late afternoon (every 12 ± 4 h) for an additional 6 days (D3–D8). Phages were stored separately and mixed at bedside before administration. All patients received co-trimoxazole 800/160 mg orally twice daily for 3 days starting on the final day of PT (D8–D10), and the two patients who opted to undergo FMT received additional vancomycin 250 mg orally every 6 h for 3 days (D9–D11) as a pre-treatment to improve FMT engraftment. After a 1-day wash-out, patients received FMT via orally administered capsules (*n* = 30) given over 2–3 h (D12).

### Bacterial culture and cryostocking

All *E. coli* strains were grown at 37 °C with agitation in Luria–Bertani (LB) broth and kept on LB agar or UriSelect plates. For long-term storage, strains were maintained as glycerol stocks containing 20% (v/v) glycerol at −80 °C. Laboratory *E. coli* strains BL21 (NEB), clinical isolate EC20 and patient strains were used for in vitro assessments of the phages and the analysis of clinical samples.

### Phage susceptibility assays

#### Dilution spot assay

For dilution spot assays, 250 µl of stationary-phase bacterial cultures (BL21, Ec20 or the patient *E. coli* strain) was mixed with 13 ml of molten LB agar (medium components (w/v) per litre: 1.0% tryptone, 0.75% NaCl, 0.5% yeast extract, 1% glucose and 0.4% agar) supplemented with CaCl_2_ (10 mM) and MgSO_4_ (2 mM), equilibrated at 55–60 °C and overlaid onto a (120 × 120 × 17 mm) square LB agar plate. Once the agar solidified, serial dilutions (3–5 µl) of phage prepared in 1× phosphate-buffered saline (PBS) were spotted onto the bacterial lawns, incubated for 18 h at 37 °C and then inspected for plaque formation. Phage concentrations were determined by counting PFUs and calculating the titre based on the dilution factor.

#### Turbidity reduction assay

For liquid phage susceptibility assays, overnight (ON) bacterial cultures were diluted 1:100 and incubated for ~2 h at 37 °C to restart growth. The bacterial cultures were adjusted to an optical density (OD_600_) of ~0.05 in LB. Individual phages were used at a final concentration of 1 × 10^8^ PFU ml^−^^1^, and the phage cocktail was an equimolar mix at a final concentration of 2 × 10^8^ PFU ml^−^^1^ in 1× PBS, corresponding to a multiplicity of infection (MOI) of 10 for turbidity reduction assays. In a 96-well plate, 160 µl medium, 20 µl bacteria and 20 µl phage suspension were combined per well. Growth controls received 20 µl of bacteria and 20 µl of PBS instead of phage, and negative controls received 20 µl of PBS and 20 µl of phage. Plates were sealed with a Breathe-Easy sealing membrane (Diversified Biotech) and incubated in a BioTek LogPhase 600 plate reader, with OD_600_ measured every 40 min for 24 h. The percentage of inhibition (PI) was calculated based on the area under the curve (AUC) of OD_600_ between 0 h and either 5 h or 18 h post-infection. PI was calculated as follows: PI = 100 × (AUC growth control − AUC phage)/AUC growth control.

### Preparation of experimental treatments (phage cocktail and FMT)

#### Phage cocktail

A two-phage cocktail consisting of two wild-type *E. coli* phages, E2 (accession OL870316) and phi41S, belonging to the genera *Tequatrovirus* and *Phapecoctavirus*, respectively, was used based on complementary host range and different receptors. Both phages were isolated from environmental wastewater sources in Switzerland and were purified for these treatments using caesium chloride density gradient ultracentrifugation. In brief, ON cultures of bacterial host strains were diluted 1:100 in LB and grown to an OD_600_ of 0.5 at 37 °C and 75 r.p.m. Phages were added at an MOI of 0.2–0.5, and the cultures were incubated for another 5 h under the same conditions. Following lysis, the cultures were centrifuged at 4 °C for 30 min at 10,000 × *g*, and the supernatants were passed through a 0.22 µm pore-size filter to remove bacterial debris. Two volumes of the clarified phage-containing supernatant were mixed with one volume of precipitation buffer (30% w/v PEG 8000, 3 M NaCl) and incubated ON at 4 °C. Phage particles were pelleted by centrifugation at 4 °C for 30 min at 10,000 × *g*, and the resulting pellet was resuspended in SM buffer. The phage suspension was purified via caesium chloride isopycnic centrifugation at 78,200 × *g* for 16 h at 4 °C in an Optima XE-90 ultracentrifuge^18^. Bands corresponding to purified phage were extracted using a 0.9 mm needle and dialysed 3 times against 0.9% NaCl in 20,000 molecular weight cut-off (MWCO) cassettes (ThermoScientific) over 24 h.

Quality control assessments were conducted externally and confirmed potency, sterility and low endotoxin levels (Supplementary Information 2 and 3). The total endotoxin units (EU) per dose were 411 for oral administration and 274 for IVS administration (concentration of 13.7 EU ml^−^^1^).

#### FMT

FMT products were supplied by the Cologne Microbiota Bank, an established manufacturing facility for FMT products. FMT capsules were manufactured at the Cologne Microbiota Bank using 50 g of donor stool from healthy volunteers. Healthy donors are screened on a regular basis for a broad range of potentially transmissible pathogens in both stool and blood in accordance with existing recommendations (EDQM *Guide to the Quality and Safety of Tissues and Cells for Human Application*, 5th edition, 2022). Of note, screening does include testing for specific pathogenic *E. coli* (EHEC, ETEC, EPEC, EIEC and EAggEC) and multidrug-resistant Gram-negative bacteria, but not for the presence of *E. coli* or uropathogenic *E. coli* in general. FMT capsules are produced from stool samples from screened donors in a dedicated clean room by homogenization, filtering, centrifugation and addition of glycerol as a cryoprotectant. The resulting suspension is then encapsulated in acid-resistant capsules^19^. Each treatment unit contained 30 capsules produced from 50 g stool in total. FMT products were stored at −80 °C until administration. The two FMT products used for patients 1 and 2 were derived from different donors based on product availability.

### Clinical sample preparation

Native urine was collected for bacterial identification and quantification, phage enumeration and downstream microbiome analysis. In addition to routine diagnostics, the *E. coli* microbial burden during the treatment phase was assessed by plating fresh urine samples in the research setting. Urine (1 ml) was centrifuged at maximum speed, the supernatant discarded, and the pellet resuspended in 1 ml of 1× PBS. A 100 µl aliquot of fresh urine and its tenfold serial dilutions were plated onto UriSelect4 (Bio-Rad) agar for uropathogen differentiation. After 16–20 h of incubation at 37 °C, colony formation was assessed and quantified. Before each administration, urine samples were obtained for phage detection by dilution spot assay (as described above). Five millilitres of urine was centrifuged at 1,500 × *g* for 10 min at room temperature, and the supernatant was sterilized using a syringe and a 0.22 µm filter. For microbiome analysis, 10 ml of the patients’ urine was centrifuged at 1,500 × *g* for 10 min at RT. Nine millilitres of supernatant was removed, and the pellet was resuspended in residual urine. The sample was then transferred into a cryotube and stored at −80 °C.

Vaginal, rectal and stool swabs were collected from patients for microbiome composition (eNAT swabs, Copan) and bacterial growth (eSwabs, Copan) analyses. In brief, eNAT swabs from each body site (vaginal, rectal and stool) were collected during predefined clinical visits. The tubes were vortexed vigorously, and the liquid was aliquoted into two cryotubes and stored at −80 °C. Likewise, eSwabs were processed using the same procedure, with the addition of 500 µl of 80% glycerol after vortexing. The sample was mixed by inversion, aliquoted into two cryotubes and then stored at −80 °C.

Patients’ blood samples were allowed to coagulate for 30 min (BD Vacutainer Serum Tube, BD) while upright at RT. The total blood volume was recorded, and the blood was centrifuged at 1,300 × *g* for 10 min at 4 °C to separate the serum from the cellular components. The resulting supernatant, representing the serum, was then aliquoted into 0.5 ml sample tubes and stored at −80 °C.

### Bacterial isolation and identification

Unless stated otherwise, bacterial cultures were incubated at 35 °C ± 2 °C and 7.5% CO_2_ for 16 to 20 h. Urine samples were evaluated for bacterial growth from BD Vacutainer (BD) and plated onto Columbia 5% sheep blood agar (COS; bioMérieux SA), colistin–nalidixic acid blood agar (CNA; bioMérieux) and UriSelect4 agar (URI4; Bio-Rad Laboratories)^20^. Vaginal and stool swabs were analysed exclusively for Gram-negative bacterial growth from eSwabs (Copan) on MacConkey agar plates without antibiotics (bioMérieux). Each morphology of growing colonies was subcultured on Columbia sheep blood agar plates without antibiotics (bioMérieux), again overnight at 37 °C, before identification using matrix-assisted laser desorption/ionization-time of flight (MALDI-ToF) mass spectrometry applying the MALDI Biotyper smart (Bruker Daltonics) using a 337-nm-wavelength nitrogen laser. MALDI-ToF MS is considered the gold standard in clinical microbiology for rapid microbial identification^21^. In short, all strains were spotted onto the MALDI target plate and overlaid with 1 μl of 70% formic acid. After drying at RT, spots were covered and dried with 1 μl α-cyano-4-hydroxycinnamic acid matrix, according to the manufacturer’s instructions. Each spot was read in the positive ion mode (detecting cations) with a m/z detection range of 2,000 to 20,000 Da, which is the range that captures most ribosomal proteins needed for bacterial identification. The species of each mass spectrum was identified using the MALDI Biotyper software package (MBT Compass 4.1.100.10) and the MBT reference library (version BDAL-12.0, including 11,897 species) at default parameter settings. The scoring criteria were Log(score) ≥ 2.0 for species-level identification and Log(score) ≥ 1.7 and < 2.0 for genus-level identification.

Bacterial growth was reported either semi-quantitatively (‘sporadic’ = 10^3^ to <10^4^ CFUs, ‘moderate’ = 10^4^ to <10^5^ CFUs and ‘abundant’ = 10^5^ to <10^6^ CFUs) or as numbers of colonies corresponding to the quadrant of the streak. All processes are part of the daily clinical routine of an ISO/IEC 17025-accredited diagnostic laboratory^22^.

### Genome sequencing and analysis of bacterial isolates

Clinical isolates were grown in LB medium at 37 °C. Genomic DNA was extracted, and sequencing libraries were prepared according to the manufacturer’s instructions. Sequencing was performed using Illumina MiSeq, generating paired-end reads whose quality was assessed using FastQC v0.11.9. Adapter and quality trimming were performed using Trimmomatic v0.39 at default settings and a minimum read length threshold of 30 bp (MINLEN:30). De novo genome assembly was conducted using SKESA (version 2.4.0), which was integrated into Ridom SeqSphere+ (Ridom GmbH, client version 10.5.5).

Virulence gene content was determined using the *E. coli* virulence factor database (version 2020-Feb-28), which was integrated into the Ridom SeqSphere+ software. Antimicrobial resistance determinants were identified using NCBI AMRFinderPlus version 4.0.15. For this characterization, core genome MLST was performed in Ridom SeqSphere+ using the core genome MLST scheme from EnteroBase (http://enterobase.warwick.ac.uk), which is based on 2,513 target genes. Genomic analyses for phylogenetic assessment were performed using the IMMENSE pipeline (version 1.2.0; https://pubmed.ncbi.nlm.nih.gov/39446690/, which is based on NextFlow version 24.04.3). Input reads were quality trimmed using Trimmomatic v0.39 with default parameters, applying a sliding window of 4:12 and a minimum read length of 100. Draft genomes were assembled using Unicycler v0.5.0 at default settings and subsequently annotated using Bakta v1.9.3. Typing was conducted using pyMLST v2.1.6. Minimum spanning trees were generated using the MBioSEQ Ridom Typer and based on a core genome of 2,513 alleles.

### Shotgun metagenomics and microbiome analysis

Genomic DNA from urine and vaginal samples was isolated using the ZymoBIOMICS DNA Microprep Kit and from stool samples using the ZymoBIOMICS DNA Miniprep Kit (both Zymo Research) following the manufacturer’s instructions, and the amount of double-stranded DNA was quantified using the Qubit dsDNA kit (Thermo Fisher Scientific). Shotgun metagenomic sequencing was performed by an external sequencing provider (Biomarker Technologies (BMK) GmbH) on a BGI platform using 2 × 150 bp paired-end reads (PE 150), generating an average of 33 million read pairs per sample after quality filtering, corresponding to approximately 10 Gb of sequencing data per sample.

To ensure consistent processing, all samples were subjected to an additional quality filtering and host read removal step using KneadData (v0.12.0), despite the initial delivery of pre-filtered reads. Trimming was performed using Trimmomatic (v0.39; SLIDINGWINDOW:4:20 MINLEN:50), and host reads were removed using Bowtie2 (v2.4.2) and the human reference genome (hg37_v0.1). Taxonomic profiling was done using MetaPhlAn 4 (v4.1.1, mpa_vJan25_CHOCOPhlAnSGB_202503). Statistical analyses were carried out using R for Statistical Computing (version 4.5.1, R Foundation for Statistical Computing). For this purpose, metagenomic data were imported using the phyloseq (v1.52.0) R package.

The visualization of the microbiome’s composition was realized using principal coordinate analysis based on the Bray–Curtis beta diversity.

### Phage–antibiotic synergy

ON bacterial cultures were diluted 1:100 into fresh LB medium and then grown at 37 °C for ~2 h to reach the logarithmic phase. The optical density of the log-phase culture was measured and adjusted to achieve a final concentration of ~1 × 10^7^ CFU ml^−^^1^. For phage preparation, individual phages were mixed to generate a phage cocktail with a final concentration of 2 × 10^9^ PFU ml^−^^1^. To examine bacteriophage–antibiotic interactions, sterile 96-well microtitre plates were prepared by adding 100 µl of the bacterial culture, 50 µl of the phage cocktail and 50 µl of co-trimoxazole solution to each well. Each plate was prepared in a way that antibiotic concentrations varied twofold (128–0.25 µg ml^−^^1^) in columns and phage MOI (100–0.0001) varied tenfold (5 × 10^8^–10^2^ PFU ml^−^^1^) in rows. For each assay, one column contained only phage, whereas one row contained only antibiotics. Controls were performed for bacterial growth (no phage or antibiotic) and medium sterility. Plates were sealed, and synergy testing was performed using a BioTek LogPhase 600 plate reader in which the OD_600_ was measured every 40 min for 24 h with shaking.

The AUC was used to measure total bacterial growth over 24 h, calculated from the raw OD time-course data using trapezoidal integration, which sums the area between consecutive OD measurements while accounting for the time between readings. Synergy was assessed using a Highest Single Agent model based directly on AUC outcomes. Interactions were classified by comparing the AUC with the best-performing single agent, applying a ±10% threshold of the single-agent AUC to define the outcomes as ‘additive’ (>10%), ‘no difference’ (within ±10%) or ‘antagonistic’ (<10%).

All data processing and visualization were conducted in R (version 4.3.2). Systematic calculations across experimental conditions were performed using the dplyr and tidyr packages. Visualizations were generated using ggplot2 with custom gradient and categorical colour mappings for the heat maps, and the results were programmatically compiled into reports using the officer and rvg packages.

### Serum neutralization

Serum samples were heat inactivated at 56 °C for 30 min. For the neutralization assay, serum samples were diluted to a final concentration of 1:10 in SM buffer and incubated with 1 × 10^9^ PFU ml^−^^1^ of phage for 1 h at 37 °C. Control reactions without serum were included in parallel. After incubation, the reactions were subjected to spot assays using tenfold serial dilutions in technical replicates to assess the residual phage activity.

### Data visualization and analysis

All statistical analyses were conducted using R for Statistical Computing (version 4.0 or higher, R Foundation for Statistical Computing). Data manipulation and visualization were performed using the tidyverse package suite for comprehensive data processing, readxl for file import, ggplot2 for statistical graphics, dplyr for data manipulation and tidyr for data reshaping. Additional packages included pracma for numerical integration, viridis for colour scales and officer with rvg for vectorized data export. Serum neutralization assays compared baseline with 16-week neutralization capacity as an unpaired baseline–last-visit comparison.

### Reporting summary

Further information on research design is available in the Nature Portfolio Reporting Summary linked to this article.

## Supplementary information

Supplementary Information Supplementary Information 1–5.

Reporting Summary

Peer Review File

Supplementary Table 1 Antibiotic susceptibility testing (AST) results for patient strains isolated from the urine pre-treatment and at follow-up (FU) visits. The time of follow-up isolation is indicated in days (D) after the start of phage therapy.

Supplementary Table 2 Virulence factor detection in sequenced isolates from patients before or after treatment.

## Source data

Source Data Fig. 2 Sheet “F2a”: sample, day, visit, and raw bacterial counts. Sheet “F2b”: sample, day, visit, and raw phage counts. Sheet “F2c”: frequency of UTI per year pre- and post-treatment.

Source Data Extended Data Tab. 1 Editable format of Extended Data Table 1 without merged cells or colors

Source Data Extended Data Fig. 1 Sheet “EDF1a-layout”: 96-well plate layout. Sheet “EDF1a-readout”: unprocessed OD600 measurements. Sheet “EDF1b”: full image of plate cropped for spot assays.

Source Data Extended Data Fig. 2 Sheet “EDF2-layout”: 96-well plate layout. Sheet “Patient1”: unprocessed OD600 measurements for patient 1 strain. Sheet “Patient2”: unprocessed OD600 measurements for patient 2 strain. Sheet “Patient3”: unprocessed OD600 measurements for patient 3 strain.

Source Data Extended Data Fig. 5 Raw phage counts per sample and bacterial host strain.

Source Data Extended Data Fig. 6 Raw phage counts per sample at indicated time point.

Source Data Extended Data Fig. 7 Sheet “EDF7-layout”: 96-well plate layout. Sheet “Patient2-D1”: unprocessed OD600 measurements for patient 2 D1 strain. Sheet “Patient2-D6”: unprocessed OD600 measurements for patient 2 D6 strain.

## Data Availability

Anonymized clinical data from the three patients included in this study were deposited in Phagistry (www.phagistry.org). Due to ethical considerations and the risk of re-identification, individual-level clinical data are not publicly available; requests for scientific use can be directed to the corresponding author. Raw metagenomic and bacterial sequencing data generated in this study have been deposited in the NCBI Sequence Read Archive (SRA) under accession number PRJNA1331746. Source data underlying the microbiological analyses, including experimental datasets used for data processing and figure generation, are accessible from source data files and have been made publicly available via GitHub and archived in Zenodo at 10.5281/zenodo.19917704 (ref. ^23^). Source data are provided with this paper.

## Extended data

**Extended Data Table 1:** Microbiological detection in clinical samples

		Urine		Vaginal		Rectal/Stool	
Patient	Visit	*E. coli* (CFU/mL)	Other (CFU/mL)	*E. coli*	Other	*E. coli* °	Other (relevant)
Patient 1	Pre-treatment	Y	N	NA	NA	Y	N
	SOT - D1	1.00E+02	*Staphylococcus capitis* (3E2)	N	N	Y	N
	D3	8.00E+01	*Staphylococcus* spp	N	N	Y	N
	EOT - D8 (pre Abx)	N	N	N	N	Y, sporadic	N
	FU1 - D12	N	N	N	N	NA	NA
	FU2 - D19	Y	N	Y	K. aerogenes	NA	NA
	FU3 - D42	N	*E. faecalis*	N	N	NA	NA
Patient 2	Pre-treatment	Y	N	NA	NA	Y	*Citrobacter* spp, *Enterococcus* spp, and *Enterobacter* spp
	SOT - D1	5.20E+07	N	Y	N	Y	N
	D3	9.30E+04	N	Y, low	N	Y, low	N
	EOT - D8 (pre Abx)	N	*Citrobacter freundii* (2E1)	N	*Citrobacter freundii*	N	*K. pneumoniae*
	FU1 - D12	N	N	N	Lacticaseibacillus rhamnosus	NA	NA
	FU2 - D19	N	N	Y	N	NA	NA
	FU3 - D42	Y	*E. faecalis*	Y	N	NA	NA
Patient 3	Pre-treatment	Y	N	Y	*Enterococcus faecalis*; *Limosilactobacillus fermentum*	Y	*Citrobacter* spp and *Enterobacter* spp
	SOT - D1	1.20E+07	*Enterobacter cloacae* complex (6E1)	Y, sporadic	N	Y	N
	D3	2.00E+06	N	N	N	Y	*Enterobacter cloacae* complex
	EOT - D8 (pre Abx)	6.50E+02	*Enterobacter cloacae* complex (7.4E3)	N	*Enterobacter cloacae* complex, low	Y	*Enterobacter cloacae* complex
	FU1 - D12	Y	N	N	*Lactobacillus crispatus*	NA	NA
	FU2 - D19	Y	N	Y	N	NA	NA
	FU3 - D42	NA	NA	NA	NA	NA	NA

* notable colony morphology change in urine isolates; ° All E. coli; ¨suscepted, not MALDI confirmed; SOT Start of therapy; D# Day of treatment; EOT End of therapy; FU Follow-up; Abx antibiotic therapy; Y Yes; N No; NA Not available; CFU/mL Colony-forming units per mililiter.

Bacterial species found in the urine or from vaginal swabs and rectal swabs/stool from patient samples pretreatment, throughout treatment, and during follow-up (FU) with the day (D) of detection after PT start. Quantification (CFU/mL) shown where available. One sample per sample type was obtained from patients at the specific timepoints.

Source data

## Code availability

Custom R scripts used for data processing, statistical analysis and figure generation are available via GitHub and archived in Zenodo at 10.5281/zenodo.19917704 (ref. ^23^).
