# Supplementary material for: Combined phage therapy and faecal microbiota transplantation to treat recurrent urinary tract infection: a case series
Source: Nat Microbiol. 2026 Jul 30;11(8):2112–8. doi: 10.1038/s41564-026-02409-0 (PMC13423874; doi:10.1038/s41564-026-02409-0)
Supplement: Supplementary file 1 — Supplementary Information 1–5. [file 41564_2026_2409_MOESM1_ESM.pdf]

nature microbiology

Supplementary information

https://doi.org/10.1038/s41564-026-02409-0

Combined phage therapy and faecal microbiota transplantation to treat recurrent urinary tract infection: a case series

In the format provided by the authors and unedited

Supplementary Information 1: Detailed patient vignettes detailing medical history and past treatments

Patient 1

Demographics:
- Female, age range 30-40 years

Clinical History:
- Sporadic urinary tract infections (UTIs) since age 13
- Marked increase in UTI frequency over the past 10 years, temporally associated with repeated dental surgeries, jawbone inflammation, and prolonged antibiotic exposure (clindamycin, amoxicillin/clavulanic acid)
- Chronic recurrent UTIs for 6 years, with symptomatic episodes every 2–4 weeks, often recurring shortly after antibiotic cessation
- 5 antibiotic courses for UTI within the last 12 month, but 10 episodes in total

Previous Treatments:
- Canephron®, Cystinol®, cranberry-based supplements, D-mannose, methenamine hippurate, chondroitin-sulfate and hyaluronic acid instillations, immunostimulatory therapy (Strovac®, Uro-Vaxom®, Uromune®), repetitive short term antibiotics, repetitive short term antibiotics, long-term antibiotic prophylaxis, vaginal estriol, and progesterone

Current Medication:
- Canephron®, Cystinol®, cranberry supplements, D-mannose, vaginal estriol, and progesterone

Symptoms:
- Severe dysuria during acute flares; mitigated urgency with high fluid intake (3–5 L/day). Flare-ups correlate with the premenstrual phase, cold exposure, fatigue, intake of sugar, histamine-rich, or iron-rich foods

Lower Urinary Tract Function:
- Normal

Bowel Function:
- Dysbiosis history; improved gastrointestinal symptoms post-fecal microbiota transfer one year prior

Risk Factors:
- Vaginal atrophy, intestinal dysbiosis (leaky gut), suspected immunosuppression (frequent HSV reactivations, chronic fatigue), and hormonal fluctuations

Comorbidities:
- Chronic dental infections, HSV reactivations, iron deficiency (possible Chlamydophila pneumoniae link), leaky gut syndrome

Diagnostic Workup:
- Vitals: Blood pressure (BP) 113/81 mmHg, heart rate (HR) 81 bpm, temperature (T(37.0 °C
- Ultrasound: Normal kidneys; bladder with 40 mL post-void residual
- Uroflowmetry: Voided volume 630 mL, Qmax 42 mL/s, bell-shaped curve
- Cystoscopy: Normal
- 3-day bladder diary: Avg. micturition volume Ø 380 mL (range 50 - 760, n = 34); daytime frequency 9 - 13x, nighttime frequency 0 - 1x; avg. fluid intake Ø 2,870 mL/24h; no urgency or incontinence episodes
- 7-day bowel diary: Number of bowel movements 11; stool volume (scale: 0 = none, 4 = high) avg. 2.5 (range 1–4); stool form (Bristol Stool Chart) avg. 5.5 (range 2–6); sensation of incomplete evacuation: 60%; no incontinence
- Urinalysis: pH 5.5, nitrite negative, leukocytes 2/µL, erythrocytes 3/µL, bacteria 13/µL
- Urine Culture: No bacterial growth (most recent); latest positive: E. coli cultivated from urine 2 months prior treatment (during symptomatic UTI)

- Questionnaires:
    - ACSS: Typical symptoms 4, Differential 2, Quality of life 4
    - IPSS: Symptoms 11; Quality of life 6
    - SF-36: Physical functioning 95%, Role limitations due to physical health 50%, Role limitations due to emotional problems 67%, Energy/fatigue 15%, Emotional well-being 28%, Social functioning 25%, Pain 12.5%, General health 20%, Health change 50%

Allergies:
- None

Patient 2

Demographics:
- Female, age range 60-70 years

Clinical History:
- First UTIs already in childhood, recurrent UTIs since young age (24 years)
- UTI episodes often associated with sexual intercourse
- Starting in 2021, UTI episodes were more frequent
- Status post pyelonephritis episodes
- Both antibiotic and non-antibiotic therapies failed to induce long-term remission
- 4 antibiotic courses for UTI within the last 12 months

Previous Treatments:
- Canephron®, Cystinol®, cranberry-based supplements, D-mannose, immunostimulatory therapy (Strovac®), repetitive short term antibiotics, vaginal estriol, traditional chinese medicine, Forskolin

Current Medication:
- Ramipril, D-mannose, VitaFemin

Symptoms:
- Dysuria, vaginal burning, cloudy and odorous urine, occasional incontinence

Lower Urinary Tract Function:
- Normal

Bowel Function:
- Normal

Risk Factors:
- None identified

Comorbidities:
- Hypertension

Diagnostic Workup:
- Vitals: BP 128/86 mmHg, HR 79 bpm, T 36.8 °C
- Ultrasound: Normal kidneys and bladder
- Uroflowmetry: Voided volume 395 mL, Qmax 32 mL/s, no residual, bell-shaped curve
- Cystoscopy: Normal
- 3-day Bladder diary: Avg. micturition volume Ø 415 mL (range 170 - 750, n = 21); daytime frequency 5 - 7x, nighttime frequency 1x; avg. fluid intake Ø 3,000 mL/24h; no urgency or incontinence episodes
- 7-day bowel diary: Number of bowel movements 12; stool volume (scale: 0 = none, 4 = high) avg. 2.5 (range 1–4); stool form (Bristol Stool Chart) avg. 3 (range 1–4); sensation of incomplete evacuation 65%; no incontinence.

- Urinalysis: pH 6.0, nitrite negative, leukocytes 155/µL, erythrocytes 7/µL, bacteria >100,000/µL
- Urine Culture: E. coli >10^5/mL, fluoroquinolone-resistant
- Questionnaires:
  - ACSS: Typical symptoms 3, Differential 1, Quality of life 1
  - IPSS: Symptoms 8; Quality of life 4
  - SF-36: Physical functioning 90%, Role limitations due to physical health 100%, Role limitations due to emotional problems 100%, Energy/fatigue 75%, Emotional well-being 76%, Social functioning 88%, Pain 58%, General health 75%, Health change 100%

Allergies:
- Dust mites; facial flushing with penicillin and doxycycline

Patient 3

Demographics:
- Female, age range 20-30 years

Clinical History:
- Recurrent E. coli UTIs for 5 years with increasing frequency.
- 5 antibiotic courses for UTI within the last 12 month

Previous Treatments:
- D-mannose, traditional Chinese medicine (TCM), and immunostimulatory therapy (Strovac), repetitive short term antibiotics, long-term antibiotic prophylaxis

Current Medication:
- None

Symptoms:
- Dysuria, urgency, foul-smelling/cloudy urine, occasional macroscopic hematuria

Lower Urinary Tract Function:
- Sporadic urgency symptoms

Bowel Function:
- Normal

Risk Factors:
- Incomplete bladder emptying; hypercapacity bladder volumes

Comorbidities:
- None

Diagnostic Workup:
- Vitals: BP 121/78 mmHg, HR 80 bpm, T 35.9 °C
- Ultrasound: Normal kidneys; post void residual 150 mL
- Uroflowmetry: Voided volume 465 mL, Qmax 43 mL/s, residuals 150 mL (first), 45 mL (second), bell-shaped curve
- Cystoscopy: Normal
- 3-day Bladder diary: Avg. micturition volume Ø 425mL (range 140 - 1250, n = 21); daytime frequency 5 - 7x, nighttime frequency 0 - 1x; avg. fluid intake Ø 2930 mL/24h; no urgency or incontinence episodes
- 7-day bowl diary: Number of bowel movements 12; stool volume (scale: 0 = none, 4 = high) avg. 2.2 (range 1–3); stool form (Bristol Stool Chart) avg. 4.8 (range 4–6); sensation of incomplete evacuation in 92%; no incontinence
- Urinalysis: pH 7.0, nitrite positive, leukocytes 1/µL, erythrocytes 1/µL, bacteria 95,131/µL Urine Culture: E. coli 10^5/mL; resistant to aminopenicillins, fluoroquinolones
- Questionnaires:
  - ACSS: Typical symptoms 8, Differential 0, Quality of life 1
  - IPSS: Symptoms 6; Quality of life 1

    - SF-36: Physical functioning 100%, Role limitations due to physical health 100%, Role limitations due to emotional problems 100%, Energy/fatigue 75%, Emotional well-being 88%, Social functioning 100%, Pain 90%, General health 85%, Health change 50%

Allergies:

- None

Supplementary Information 2: Certificate of analysis for Bat001 (phage phi41S)

sciensano

Sonja Milek
Universität Zürich
Forschung Neuro-Urologie   Balgrist Campus
Lengghalde 5
8008 Zürich
SWITZERLAND

Test Report S23BD02796

Report Date: 02/05/2023

Version 01 (English)   Your Reference: Bat001

In case of questions about this report please contact:

Pieter-Jan Ceyssens (email: Pieter-Jan.Ceyssens@sciensano.be - tel:)
Wesley Mattheus (email: wesley.mattheus@sciensano.be - tel: +32 (0)2/373 32 24)

Sample Information

Sciensano Sample Ref.:	S23BD02796	Sample Type:	
Client Sample Ref.:	**Bat001**	Sample State:	
		Received Date:	05/04/2023

Lab: Bacteriophage QC

Phage Identification

Start: 02/05/2023 End: 02/05/2023 SOP / Method of Analysis: -

Parameter	Result
Phage Identification<br>*Confirmed using Illumina MiSeq; From 2,044,818 reads, 99.62% was phage derived. A phiV205-1 related induced prophages was detected at low abundance (0.003%). Only a single SNP with frequency >1% was detected between both 41S batches (77735 T_C, 1.24%).*	Escherichia phage 41S

Lab: Medicines

Microbial contamination

Start: 21/04/2023 End: 26/04/2023 SOP / Method of Analysis: Ph Eur monograph

Parameter	Result
TAMC	Conform
TYMC	Conform

Lab: Biochemistry Vaccine

Endotoxin phages by rFC

Start: 26/04/2023 End: 26/04/2023 SOP / Method of Analysis: SOP 42/III-115

Parameter	Result	Units
Endotoxins Phages	<5	EU/mL

Attachment 1: 230316-124058-F23BD01926.pdf

Supplementary Information 3: Certificate of analysis for Bat003 (phage E2)

sciensano

Sonja Milek
Universität Zürich
Forschung Neuro-Urologie		Balgrist Campus
Lengghalde 5
8008 Zürich
SWITZERLAND

Test Report S23BD03576

Report Date: 03/05/2023

Version 01 (English)

Your Reference: Bat003

In case of questions about this report please contact:

Pieter-Jan Ceyssens (email: Pieter-Jan.Ceyssens@sciensano.be - tel:)

Wesley Mattheus (email: wesley.mattheus@sciensano.be - tel: +32 (0)2/373 32 24)

Sample Information

Sciensano Sample Ref.:	S23BD03576	Sample Type:	
Client Sample Ref.:	Bat003	Sample State:	
		Received Date:	02/05/2023

Lab: Medicines

Microbial contamination

Start: 21/04/2023 End: 28/04/2023 SOP / Method of Analysis: Ph Eur monograph

Parameter	Result
TAMC	Conform
TYMC	Conform

Lab: Biochemistry Vaccine

Endotoxin phages by rFC

Start: 26/04/2023 End: 26/04/2023 SOP / Method of Analysis: SOP 42/III-115

Parameter	Result	Units
Endotoxins Phages	112,3	EU/mL

Remarks / interpretation: *test réalisé par ZB, réintroduit dans le LIMS par FLA*

Above results relate only to the tested samples identified on the report. This test report shall not be reproduced except in full, without written approval of the head of the laboratory. Sampling of the above identified samples was not performed by the laboratory. The (technical) measurement uncertainty of the results may be communicated on request by the contact person. The opinions/interpretations expressed in this report are outside the scope of the MJA attestation of this OMCL.

[2] Identified results were modified since the previous version of the report. The new results replace the previously reported results.

[3] Result obtained via outsourcing. The code of the outsourced laboratory is shown on the report above the results. The complete identification of the laboratory can be obtained upon request from Sciensano.

APPROVED
By Pieter-Jan Ceyssens on 03/05/2023 13:55:54
Lab Supervisor

Supplementary Information 4: Patient perspectives

Patients were contacted via email and asked to provide their perspective on the treatment(s) they received after 24 months. Patient responses are provided in the originally corresponded language and any change to the original text is indicated in ().

Patient 1

(original, English): "Treatment procedures: The phage treatment required a more invasive treatment as compared to oral antibiotics since phages had to be instilled into the bladder several times a day. Instillations were however tolerable very well. Additional oral phage administration went very smoothly. Sterile conditions were of utmost importance for phage instillations. At first glance treatment cost may appear higher than for antibiotics due to special equipment and more medical assistance needed.
Fecal microbiota transplantation by capsules to be swallowed went very smoothly and was neither invasive nor unpleasant. Outcomes: Phage therapy was absolutely worth it: I had chronic bladder inflammation for 10 years with recurrent acute phases every 2-4 weeks meaning my life was centered around the bladder, restricting diet, clothes, traveling, job, partner-relationship. Now I live a normal life: It is as if I have a new life. In sum, the cost related to antibiotic treatments and other treatment ideas amounted to sums far higher than the cost related to phage therapy. Phage therapy should become a standard treatment in our Western societies.
I consider the same to be true for fecal transplantation: After many many courses of antibiotics, I had Clostridium Difficile, many digestion problems, abdominal pain and a transparent urine, all showing that my microbiome had taken a serious hit. Taking commercial pro- and prebiotics did not change this. After the first fecal transplantation, normal yellow urine colour returned and digestion and abdominal problems were resolved."

Patient 2

(original, English): "What do you think I have felt after years of increasing despair, after all the experiences with incompetent, indifferent and helplessness specialists, nearly constant pains, restrictions and renunciation in daily life, when after the first intake of a phage cocktail my urine becomes in a sudden totally clear and free of germs? And so was the next day and the following days during the treatment in Zurich - until now. You can imagine: it was a turning-point in my life!
I experienced (t)his therapy firsthand. It was and is still effective and successful and for patients simply applicable without any side effects. Therefore I strongly advocate to support the scientific research and the scientific recognition of(ŧ) the phage therapy, because this therapy gives legitimate hope to all suffering people for a complete recovery."

Patient 3

(original, English): “Following phage therapy in June 2023, my symptoms have improved considerably to date. Before the treatment, I repeatedly had symptoms typical of cystitis. During the therapy, my symptoms disappeared completely for the first time since they had started. Consciously monitoring how much fluid I drank and how much urine I passed was also very helpful. After the phage therapy was finished, I still had sporadic intervals with symptoms typical of cystitis, but these were weaker, and each lasted only one or two days. About a year after treatment, the symptoms subsided even further. Today, about two and a half years after the treatment, I am practically symptom-free the whole time. In summary, the symptoms typical of cystitis completely subsided during therapy, recurred regularly in the following year, albeit considerably less frequently and with reduced severity, and have become fewer and weaker since then. In conclusion, I personally benefited greatly from the therapy in the long term.”

Supplementary Information 5: CARE Checklist

CARE case report guidelines — **CARE Checklist of information to include when writing a case report**

(cc) BY-NC-ND

Topic	Item	Checklist item description	Reported on Line
Title	1	The diagnosis or intervention of primary focus followed by the words "case report"	2
Key Words	2	2 to 5 key words that identify diagnoses or interventions in this case report, including "case report"	32-33
Abstract (no references)	3a	Introduction: What is unique about this case and what does it add to the scientific literature?	36-38
	3b	Main symptoms and/or important clinical findings	36-38
	3c	The main diagnoses, therapeutic interventions, and outcomes	38-40
	3d	Conclusion—What is the main "take-away" lesson(s) from this case?	40-41
Introduction	4	One or two paragraphs summarizing why this case is unique (**may include** references)	47-52
Patient Information	5a	De-identified patient specific information.	Supp Info 1
	5b	Primary concerns and symptoms of the patient.	54-59
	5c	Medical, family, and psycho-social history including relevant genetic information	Supp Info 1
	5d	Relevant past interventions with outcomes	Supp Info 1
Clinical Findings	6	Describe significant physical examination (PE) and important clinical findings.	Supp Info 1
Timeline	7	Historical and current information from this episode of care organized as a timeline	Figure 1
Diagnostic Assessment	8a	Diagnostic testing (such as PE, laboratory testing, imaging, surveys).	61-65
	8b	Diagnostic challenges (such as access to testing, financial, or cultural)	na
	8c	Diagnosis (including other diagnoses considered)	na
	8d	Prognosis (such as staging in oncology) where applicable	na
Therapeutic Intervention	9a	Types of therapeutic intervention (such as pharmacologic, surgical, preventive, self-care)	67-71
	9b	Administration of therapeutic intervention (such as dosage, strength, duration)	Fig 1; 256-267
	9c	Changes in therapeutic intervention (with rationale)	na
Follow-up and Outcomes	10a	Clinician and patient-assessed outcomes (if available)	131-136; Fig 2C
	10b	Important follow-up diagnostic and other test results	Supp Table 2; Fig 2C
	10c	Intervention adherence and tolerability (How was this assessed?)	80-81
	10d	Adverse and unanticipated events	80
Discussion	11a	A scientific discussion of the strengths AND limitations associated with this case report	139; 148-149
	11b	Discussion of the relevant medical literature **with references**.	140-146
	11c	The scientific rationale for any conclusions (including assessment of possible causes)	151-165
	11d	The primary "take-away" lessons of this case report (without references) in a one paragraph conclusion	170-176
Patient Perspective	12	The patient should share their perspective in one to two paragraphs on the treatment(s) they received	Supp Info 2
Informed Consent	13	Did the patient give informed consent? Please provide if requested	Yes ☒ No ☐
