# Supplementary material for: Combined phage therapy and faecal microbiota transplantation to treat recurrent urinary tract infection: a case series
Source: Nat Microbiol. 2026 Jul 30;11(8):2112–8. doi: 10.1038/s41564-026-02409-0 (PMC13423874; doi:10.1038/s41564-026-02409-0)
Supplement: Supplementary file 2 — Reporting Summary [file 41564_2026_2409_MOESM2_ESM.pdf]

nature portfolio

Corresponding author(s): Shawna McCallin

Last updated by author(s): 2026.04.30

Reporting Summary

Nature Portfolio wishes to improve the reproducibility of the work that we publish. This form provides structure for consistency and transparency in reporting. For further information on Nature Portfolio policies, see our Editorial Policies and the Editorial Policy Checklist.

Statistics

For all statistical analyses, confirm that the following items are present in the figure legend, table legend, main text, or Methods section.

n/a	Confirmed	
☐	☒	The exact sample size (*n*) for each experimental group/condition, given as a discrete number and unit of measurement
☐	☒	A statement on whether measurements were taken from distinct samples or whether the same sample was measured repeatedly
☐	☒	The statistical test(s) used AND whether they are one- or two-sided<br>*Only common tests should be described solely by name; describe more complex techniques in the Methods section.*
☒	☐	A description of all covariates tested
☒	☐	A description of any assumptions or corrections, such as tests of normality and adjustment for multiple comparisons
☐	☒	A full description of the statistical parameters including central tendency (e.g. means) or other basic estimates (e.g. regression coefficient) AND variation (e.g. standard deviation) or associated estimates of uncertainty (e.g. confidence intervals)
☐	☒	For null hypothesis testing, the test statistic (e.g. *F*, *t*, *r*) with confidence intervals, effect sizes, degrees of freedom and *P* value noted<br>*Give P values as exact values whenever suitable.*
☒	☐	For Bayesian analysis, information on the choice of priors and Markov chain Monte Carlo settings
☒	☐	For hierarchical and complex designs, identification of the appropriate level for tests and full reporting of outcomes
☒	☐	Estimates of effect sizes (e.g. Cohen's *d*, Pearson's *r*), indicating how they were calculated

Our web collection on statistics for biologists contains articles on many of the points above.

Software and code

Policy information about availability of computer code

Data collection	REDCap project specific database
Data analysis	SKESA, SeqSphere+ softwares. Custom code was written in Nextflow or R using publicly available tools

For manuscripts utilizing custom algorithms or software that are central to the research but not yet described in published literature, software must be made available to editors and reviewers. We strongly encourage code deposition in a community repository (e.g. GitHub). See the Nature Portfolio guidelines for submitting code & software for further information.

Data

Policy information about availability of data

All manuscripts must include a data availability statement. This statement should provide the following information, where applicable:

- Accession codes, unique identifiers, or web links for publicly available datasets
- A description of any restrictions on data availability
- For clinical datasets or third party data, please ensure that the statement adheres to our policy

Anonymised clinical data from the three patients included in this study have been deposited in the Phagistry (www.phagistry.org
). Due to ethical considerations and the risk of re-identification, individual-level clinical data are not publicly available; requests for scientific use can be directed to the corresponding author. Raw metagenomic and bacterial sequencing data generated in this study have been deposited in the NCBI Sequence Read Archive (SRA) under accession number PRJNA1331746. Source data underlying the microbiological analyses, including experimental datasets used for data processing and figure

generation, are accessible from source data files and have been made publicly available on GitHub and archived in Zenodo at https://doi.org/10.5281/zenodo.19917704

Research involving human participants, their data, or biological material

Policy information about studies with human participants or human data. See also policy information about sex, gender (identity/presentation), and sexual orientation and race, ethnicity and racism.

Reporting on sex and gender	All patients were female. These were individual patients outside of a formal study where treatments are given in the best of interest of the patients, therefore gender considerations did not contribute to patient selection. Informed consent was obtained prior to treatment.
Reporting on race, ethnicity, or other socially relevant groupings	No information on these parameters was included.
Population characteristics	Three female patients between 20-70 years of age with long-standing histories of recurrent UTI (5–44 years).
Recruitment	These were individual patients outside of a formal study where treatments are given in the best of interest of the patients, therefore randomization is not applicable in this setting.
Ethics oversight	Experimental treatments/individual treatment attempts are exempt from ethical approval in Switzerland/Germany. Ethical approval was obtained from the KEK Zürich and the ethics committee of the University Hospital of Cologne for the reuse of clinical data and samples and biobanking.

Note that full information on the approval of the study protocol must also be provided in the manuscript.

Field-specific reporting

Please select the one below that is the best fit for your research. If you are not sure, read the appropriate sections before making your selection.

☒ Life sciences ☐ Behavioural & social sciences ☐ Ecological, evolutionary & environmental sciences

For a reference copy of the document with all sections, see nature.com/documents/nr-reporting-summary-flat.pdf

Life sciences study design

All studies must disclose on these points even when the disclosure is negative.

Sample size	Three
Data exclusions	One genome sequence was excluded due to too low coverage.
Replication	Experiments were performed in triplicate, either technical or biological, as indicated in the manuscript.
Randomization	These were individual patients outside of a formal study where treatments are given in the best of interest of the patients, therefore randomization is not applicable in this setting.
Blinding	These were individual patients outside of a formal study where treatments are given in the best of interest of the patients, therefore blinding is not applicable in this setting.

Reporting for specific materials, systems and methods

We require information from authors about some types of materials, experimental systems and methods used in many studies. Here, indicate whether each material, system or method listed is relevant to your study. If you are not sure if a list item applies to your research, read the appropriate section before selecting a response.

Materials & experimental systems

n/a	Involved in the study
☒	☐ Antibodies
☒	☐ Eukaryotic cell lines
☒	☐ Palaeontology and archaeology
☒	☐ Animals and other organisms
☐	☒ Clinical data
☒	☐ Dual use research of concern
☒	☐ Plants

Methods

n/a	Involved in the study
☒	☐ ChIP-seq
☒	☐ Flow cytometry
☒	☐ MRI-based neuroimaging

Clinical data

Policy information about clinical studies

All manuscripts should comply with the ICMJE guidelines for publication of clinical research and a completed CONSORT checklist must be included with all submissions.

Clinical trial registration	These were individual patients outside of a formal study where treatments are given in the best of interest of the patients, therefore it is not a formal trial and does not require registration.
Study protocol	These were individual patients outside of a formal study where treatments are given in the best of interest of the patients, therefore does not entail a formal study protocol.
Data collection	Treatments were performed in Zurich, Switzerland and Frankfurt, Germany, where samples were also collected and analysed to generate data. Patients provided informed consent for the reuse of clinical data and samples and biobanking.
Outcomes	These were individual patients outside of a formal study where treatments are given in the best of interest of the patients, therefore no formal endpoints were elaborated. Patients were monitored for the presence and concentration of bacteria in urine and for the frequency of UTI episodes and need for antibiotic treatment.

Plants

Seed stocks	*Report on the source of all seed stocks or other plant material used. If applicable, state the seed stock centre and catalogue number. If plant specimens were collected from the field, describe the collection location, date and sampling procedures.*
Novel plant genotypes	*Describe the methods by which all novel plant genotypes were produced. This includes those generated by transgenic approaches, gene editing, chemical/radiation-based mutagenesis and hybridization. For transgenic lines, describe the transformation method, the number of independent lines analyzed and the generation upon which experiments were performed. For gene-edited lines, describe the editor used, the endogenous sequence targeted for editing, the targeting guide RNA sequence (if applicable) and how the editor was applied.*
Authentication	*Describe any authentication procedures for each seed stock used or novel genotype generated. Describe any experiments used to assess the effect of a mutation and, where applicable, how potential secondary effects (e.g. second site T-DNA insertions, mosiacism, off-target gene editing) were examined.*
