# Supplementary material for: Combined phage therapy and faecal microbiota transplantation to treat recurrent urinary tract infection: a case series
Source: Nat Microbiol. 2026 Jul 30;11(8):2112–8. doi: 10.1038/s41564-026-02409-0 (PMC13423874; doi:10.1038/s41564-026-02409-0)
Supplement: Supplementary file 3 — Peer Review File [file 41564_2026_2409_MOESM3_ESM.pdf]

nature portfolio

Peer Review File

Combined Phage therapy and fecal microbiota transplantation to treat recurrent urinary tract infection: a case series

Corresponding Author: Dr Shawna McCallin

Version 0:

Reviewer comments:

Reviewer #1

(Remarks to the Author)
According to the title of the present manuscript, McCallin and colleagues share their findings from the treatment of patients with recurrent urinary tract infections using phage therapy (PT) plus fecal microbiota transplantation (FMT).

In the abstract and main text, we find out that the manuscript describes three cases, two patients treated with (semi-personalized) PT + antibiotics (cotrimoxazole and vancomycin) + FMT, and one patient treated with PT + antibiotics.

The present case series thus pertains to two patients treated with PT + antibiotics + FMT.

Questions/comments:

What is meant by semi-personalized PT?

What is the rationale behind this sequence of different treatments and why was one patient not treated with FMT? Is the treatment protocol based on an in vitro study, or on previous (historical) case studies or series? Why were PT and antibiotics applied sequentially (with 1 day overlap) instead of simultaneously.

It is stated “Unlike antibiotics, which disrupt the microbiome and provoke overgrowth of secondary pathogens, PT’s narrow specificity could preserve microbial balance, potentially reducing recurrence driven by opportunistic species”. Indeed, but it is also suggested (also by the authors) that PT alone cannot achieve sustainable eradication. So, in that context, the addition of FMT makes sense. Yet, potent antibiotics were also used after PT and continued for 24 months. Was the applied antibiotic therapy adapted (e.g. less intensive) compared to patients that do not receive PT or PT plus FMT?

It is stated that “microbial concentrations in the urine were unknown at the start of treatment (SOT)”. Why were they not determined? Yet, further in the text, it is stated that “Two of the three patients presented with high E. coli titers in the urine at the SOT”.

Not much is mentioned regarding the used phages. On what basis were these two phages chosen? What are their host ranges, bacterial receptors, interactions, activities of each phage against the patients’ E. coli strains. In other words, is there a rationale for using these two particular phages, together?,,..

For phage susceptibility testing in liquid culture, a multiplicity of infection (MOI) of 10 was chosen, which is quite high, especially for a cocktail of phages. Why was MOI 10 chosen?

Overall, the presentation and discussion of results is rather brief and sometimes incomplete (e.g., whole genome sequencing results).

Supplementary Table 2 shows that patient 3 presented with three pre-treatment isolates with different antibiotic susceptibility patterns. Was phage susceptibility tested for all three isolates? If so, can results be presented in Supplementary Figure 1? Were these three pre-treatment isolates genetically identical (were antibiotic resistance genes or mutations acquired?)?

Supplementary Table 3: a dendrogram visualizing similarity and distances between isolates would be nice.

Supplementary Figure 1 could use more info, such as start concentrations, MOIs, and dilutions. This would avoid flipping back and forth to the methods section. It would also be nice to visualize the results of phage-antibiotic interaction testing.

Reviewer #2

(Remarks to the Author)
The authors have conducted a clinical study on three patients with chronic recurrent UTIs that examines the preliminary efficacy of an experimental treatment that involves oral and systemic phage administration and antibiotic intervention. Two of the three patients then received antibiotic conditioning (vancomycin) and oral encapsulated FMT. The authors report variable clinical success of the therapeutic strategy that is to some extent disconnected from the microbiological outcomes (i.e., persistence of E. coli post intervention). While the study provides interesting data that can inform and guide future phage interventions and RCTs, many areas would benefit from further details and improved clarity. Please see my comments below in the order they appear in text.

1. It would be useful in the introduction to clarify the concept of chronic recurrent UTI (i.e., the difference between the concept of re-infection vs. the persistence of the bacterium in bladder epithelial cells and re-emergence due to some trigger).

2. L54-55. Please reference the following statement: “Alternative therapeutic strategies such as phage therapy (PT) and fecal microbiota transplantation (FMT) have been proposed to address this clinical challenge.”

3. L66-67. “Here we report our rationale…”
Please adjust to PT, antibiotics and FMT

4. L68-69. What do the authors mean by the phrase:
“in anticipation of providing in-depth sample analysis to support protocol development and preparation for a RCT.” I understand the idea of preliminary investigation for RCT design but what is the in-depth sample analysis referring to?

5. Supplementary information 1. Why is the age provided as a decade long range? What does “improved bowel function” following FMT refer to for patient 1? Also, please clarify if this is referring to the current FMT received in this study.

6. L84-85. The statement “Pre-treatment E. coli isolates were found to be genetically stable by whole genome sequencing (WGS)” requires some evidencing. If genomes are available, then a comparative ANI or k-mer analysis would help confirm this.

7. Supplementary table 1. This table would benefit from a more detailed caption for clarity.

8. L85-86. The authors state: “consistently susceptible to co-trimoxazole (Supplementary Table 2)”. I may have misunderstood something but how does this statement align with data from patient 3 that shows resistance (Feb 2023)?

9. Supplementary figure 1B. If the data presented in bacterial CFU, why do the authors see increases with increased phage cocktail levels?

10. L87-91. These lines report on a substantial amount of results with no data shown.

11. Could the authors provide a rationale for antibiotic conditioning with oral vancomycin prior to FMT if the purpose of FMT was E. coli displacement in the gut?

12. Figure 1. It would be useful to include in the figure that only 2/3 patients received FMT.

13. L118-119 / Supplementary figure 3. How does the statement align with the fact that patient 1 had higher bacterial counts at the end when compared to baseline?

14. Supplementary figure 4. What does one-sided comparisons mean? What is the specific statistical test used?

15. Supplementary table 3. This would benefit from a better representation of phylogeny. Phylogeny based on ANI, k-mer or the current MLST would be more visually informative than tabular format.

16. Supplementary figure 7. This is a PCoA not PCA, please adjust in text. Also, it is stated that this represents Bray-Curtis dissimilarity but in the methods the authors report using Jensen-Shannon divergence?

17. Figure 2C. This figure does not quite capture L173-178. Is there a way to visually represent all that is mentioned in those lines?

18. Do the authors have any information on the species strain richness of E. coli in the urinary tract? Can there only be one strain at a time, similar to that observed for B. fragilis in the gut?

19. There is emphasis on phage therapy in the discussion but to me patient 3 who was not on FMT had the worst outcome (Figure 2C) and the success of patient 2 seems attributable to strain replacement which the assumption is it is coming from the FMT donor(?)

20. As follow on from above, it would be important to look at the metagenome-assembled genome of E. coli from the donor FMT vs patient E. coli genome at baseline vs patient E. coli genome post intervention to determine if the donor contributed the less pathogenic strain that displaced the patient strain.

21. The illustrations, particularly those in supplementary material, would benefit from improved labelling, expanded legends,

larger fonts, and so on.

Online Methods

22. What is the origin of the phages used in the study?

23. The FMT appears to have originated from two separate donors based on PCoA. Please provide more details.

24. The FMT protocol cites a paper from 2015 regarding the capsules that itself cites the seminal NEJM RCT from 2011 with regard to donor screening. Donor screening protocols have been updated quite substantially since the transfer of antibiotic-resistant E. coli through FMT. Can the authors clarify the protocols used and cite more up-to-date literature if available from the bank?

Reviewer #3

(Remarks to the Author)
TThis paper addresses the treatment of recurrent urinary tract infections (rUTIs), a debilitating condition that often leads to repeated antibiotic courses and contributes to antimicrobial resistance. The concept of combining phage therapy with faecal microbiota transplantation represents a novel therapeutic approach that, if successful, could substantially improve patients' quality of life while mitigating future antibiotic resistance.
However, the findings should be regarded as preliminary given the very limited number of participants. Future studies will require much larger sample sizes to establish clinical efficacy and overall effectiveness, particularly as the treatment appeared effective in only two of the three participants. Furthermore, the study lacks a control group against which treatment effectiveness can be compared and provides limited assessment of immune responses to treatment or long term prognosis. These issues should be explicitly acknowledged as limitations within the manuscript, together with a clearer discussion of future research needs. Overall, the study would benefit from being framed more explicitly as a proof of concept pilot investigation.
Areas for authors to address
1. UTI diagnostic criteria
This study followed three individuals with recurrent UTIs. Could the authors clarify the specific diagnostic criteria used to confirm UTI? For example, was diagnosis based on:
o patient reported symptoms alone;
o microbiological findings (e.g. specified CFU thresholds); or
o a combination of clinical and laboratory parameters?
2. Phage cocktail composition
How many distinct phage strains were included in the therapeutic cocktail, and how were they selected? This information is essential for understanding the breadth of bacterial targeting and the potential for resistance development.
3. E. coli monitoring and analysis
The monitoring of urinary E. coli titres shows a gap between day 8 and day 12. As co trimoxazole administration overlaps with this period, it is difficult to disentangle the respective contributions of phage therapy and antibiotics to the observed reduction in bacterial load. More frequent quantitative cultures during and immediately after antibiotic exposure, particularly between days 8–12, would strengthen interpretation of the treatment effects and help clarify whether the observed bacterial decline can be attributed primarily to phage therapy, co trimoxazole, or their combination.
4. FMT screening and characterisation of E. coli
The manuscript states that FMT capsules were produced from healthy donors who were screened for transmissible pathogens. However, it is unclear whether the FMT material contained E. coli and, if so, how these strains were characterised. Given that many E. coli strains are commensal, whereas only specific lineages (e.g. uropathogenic E. coli) are associated with rUTI, it would be important to clarify:
o whether E. coli was present in the FMT product; and
o whether donors were screened specifically for uropathogenic E. coli strains.
5. Patient selection criteria
Only three patients were included, yet they represent a wide age range. Individuals over the age of 65 commonly have multiple comorbidities that may contribute to rUTI, making the findings more difficult to interpret. Could the authors provide detailed participant selection criteria, including explicit inclusion and exclusion criteria (with justification), age range specifications, assessment of comorbidities, and the rationale for the observed demographic heterogeneity? This information is essential for defining the target patient population, enabling replication of the study, and interpreting the clinical significance of the reported outcomes.

Decision Letter:

17th December 2025

*Please ensure you delete the link to your author homepage in this e-mail if you wish to forward it to your co-authors.

Dear Shawna,

Thank you for your patience while your manuscript "Bacteriophage therapy plus fecal microbiota transplantation to treat recurrent urinary tract infection (rUTI): a case series" was under peer-review at Nature Microbiology. It has now been seen by 3 referees, whose expertise and comments you will find at the end of this email. Although they find your work of some potential interest, they have raised a number of important concerns that will need to be addressed before we can consider publication of the work in Nature Microbiology.

In particular, all referees are concerned by the relatively small number of patients tested, and the lack of a control group. The referees ask for more information and clarification, and they say the study would need to be substantially revised to clearly frame the work as exploratory, temper claims of treatment effect, and focus on descriptive clinical and microbiological findings.

Editorially, we will also need several additional points to be addressed:

- methods don't include any information about the patients, ethics, written informed consent. This needs to be provided

- there is no Data Availability Statement and no mention of access to shotgun metagenomics. Please provide this information

- is this part of a trial? We need more information about patient selection, phage selection, why different treatments were chosen etc.

- do you have a research protocol you can provide? IRB approval?

- as this is a case series, you cannot make claims about specific outcomes like safety or efficacy. It's purely observational. You need to report in line with the CARE guidelines (the CONSORT for case reports).

Should further experimental data and text changes allow you to address these criticisms, we would be happy to look at a revised manuscript.

We are committed to providing a fair and constructive peer-review process. Please do not hesitate to contact us if there are specific requests from the reviewers that you believe are technically impossible or unlikely to yield a meaningful outcome.

We strongly support public availability of data. Please place the data used in your paper into a public data repository, if one exists, or alternatively, present the data as Source Data or Supplementary Information. If data can only be shared on request, please explain why in your Data Availability Statement, and also in the correspondence with your editor. For some data types, deposition in a public repository is mandatory - more information on our data deposition policies and available repositories can be found at https://www.nature.com/nature-research/editorial-policies/reporting-standards#availability-of-data.

Please include a data availability statement as a separate section after Methods but before references, under the heading "Data Availability". This section should inform readers about the availability of the data used to support the conclusions of your study. This information includes accession codes to public repositories (data banks for protein, DNA or RNA sequences, microarray, proteomics data etc…), references to source data published alongside the paper, unique identifiers such as URLs to data repository entries, or data set DOIs, and any other statement about data availability. At a minimum, you should include the following statement: “The data that support the findings of this study are available from the corresponding author upon request”, mentioning any restrictions on availability. If DOIs are provided, we also strongly encourage including these in the Reference list (authors, title, publisher (repository name), identifier, year). For more guidance on how to write this section please see: http://www.nature.com/authors/policies/data/data-availability-statements-data-citations.pdf

When revising your manuscript:

* Include a “Response to referees” document detailing, point-by-point, how you addressed each referee comment. If no action was taken to address a point, you must provide a compelling argument. This response will be sent back to the referees along with the revised manuscript.

* If you have not done so already we suggest that you begin to revise your manuscript so that it conforms to our Brief Communication format instructions at http://www.nature.com/nmicrobiol/info/final-submission. Refer also to any guidelines provided in this letter.

* Include a revised version of any required reporting checklist. It will be available to referees (and, potentially, statisticians) to aid in their evaluation if the manuscript goes back for peer review. A revised checklist is essential for re-review of the paper.

When submitting the revised version of your manuscript, please pay close attention to our href="https://www.nature.com/nature-portfolio/editorial-policies/image-integrity">Digital Image Integrity Guidelines. and to the following points below:

-- that unprocessed scans are clearly labelled and match the gels and western blots presented in figures.
-- that control panels for gels and western blots are appropriately described as loading on sample processing controls
-- all images in the paper are checked for duplication of panels and for splicing of gel lanes.

EXTENDED DATA FIGURES

When re-submitting your manuscript, please ensure that any supplementary figures and tables that are crucial to the manuscript’s conclusions are converted into Extended Data figures and tables to increase visibility of these data. Extended Data figures and tables are online-only (present in the online PDF and full-text HTML versions of the paper), peer-reviewed display items that provide essential background to the article but are not included in the main article due to space constraints. A maximum of ten Extended Data display items (figures and tables) is permitted.

Finally, please ensure that you retain unprocessed data and metadata files after publication, ideally archiving data in perpetuity, as these may be requested during the peer review and production process or after publication if any issues arise.

Please use the link below to submit a revised paper:

Link Redacted

Note: This url links to your confidential homepage and associated information about manuscripts you may have submitted or be reviewing for us. If you wish to forward this e-mail to co-authors, please delete this link to your homepage first.

Nature Microbiology is committed to improving transparency in authorship. As part of our efforts in this direction, we are now requesting that all authors identified as 'corresponding author' on published papers create and link their Open Researcher and Contributor Identifier (ORCID) with their account on the Manuscript Tracking System (MTS), prior to acceptance. This applies to primary research papers only. ORCID helps the scientific community achieve unambiguous attribution of all scholarly contributions. You can create and link your ORCID from the home page of the MTS by clicking on 'Modify my Springer Nature account'. For more information please visit please visit www.springernature.com/orcid.

If you wish to submit a suitably revised manuscript we would hope to receive it within 6 months. If you cannot send it within this time, please let us know.

In the meantime we hope that you find our referees' comments helpful.

Yours sincerely,

****
Reviewer Expertise:

Referee #1: Phage therapy
Referee #2: FMT, microbiota, metagenomics, sequencing
Referee #3: UTIs

Reviewer Comments:

Reviewer #1 (Remarks to the Author):

According to the title of the present manuscript, McCallin and colleagues share their findings from the treatment of patients with recurrent urinary tract infections using phage therapy (PT) plus fecal microbiota transplantation (FMT).

In the abstract and main text, we find out that the manuscript describes three cases, two patients treated with (semi-personalized) PT + antibiotics (cotrimoxazole and vancomycin) + FMT, and one patient treated with PT + antibiotics.

The present case series thus pertains to two patients treated with PT + antibiotics + FMT.

Questions/comments:

What is meant by semi-personalized PT?

What is the rationale behind this sequence of different treatments and why was one patient not treated with FMT? Is the treatment protocol based on an in vitro study, or on previous (historical) case studies or series? Why were PT and antibiotics applied sequentially (with 1 day overlap) instead of simultaneously.

It is stated "Unlike antibiotics, which disrupt the microbiome and provoke overgrowth of secondary pathogens, PT's narrow specificity could preserve microbial balance, potentially reducing recurrence driven by opportunistic species". Indeed, but it is also suggested (also by the authors) that PT alone cannot achieve sustainable eradication. So, in that context, the addition of FMT makes sense. Yet, potent antibiotics were also used after PT and continued for 24 months. Was the applied antibiotic therapy adapted (e.g. less intensive) compared to patients that do not receive PT or PT plus FMT?

It is stated that "microbial concentrations in the urine were unknown at the start of treatment (SOT)". Why were they not determined? Yet, further in the text, it is stated that "Two of the three patients presented with high E. coli titers in the urine at the SOT".

Not much is mentioned regarding the used phages. On what basis were these two phages chosen? What are their host ranges, bacterial receptors, interactions, activities of each phage against the patients' E. coli strains. In other words, is there a rationale for using these two particular phages, together?,,..

For phage susceptibility testing in liquid culture, a multiplicity of infection (MOI) of 10 was chosen, which is quite high, especially for a cocktail of phages. Why was MOI 10 chosen?

Overall, the presentation and discussion of results is rather brief and sometimes incomplete (e.g., whole genome sequencing results).

Supplementary Table 2 shows that patient 3 presented with three pre-treatment isolates with different antibiotic susceptibility patterns. Was phage susceptibility tested for all three isolates? If so, can results be presented in Supplementary Figure 1? Were these three pre-treatment isolates genetically identical (were antibiotic resistance genes or mutations acquired?)?

Supplementary Table 3: a dendrogram visualizing similarity and distances between isolates would be nice.

Supplementary Figure 1 could use more info, such as start concentrations, MOIs, and dilutions. This would avoid flipping back and forth to the methods section. It would also be nice to visualize the results of phage-antibiotic interaction testing.

Reviewer #2 (Remarks to the Author):

The authors have conducted a clinical study on three patients with chronic recurrent UTIs that examines the preliminary efficacy of an experimental treatment that involves oral and systemic phage administration and antibiotic intervention. Two of the three patients then received antibiotic conditioning (vancomycin) and oral encapsulated FMT. The authors report variable clinical success of the therapeutic strategy that is to some extent disconnected from the microbiological outcomes (i.e., persistence of E. coli post intervention). While the study provides interesting data that can inform and guide future phage interventions and RCTs, many areas would benefit from further details and improved clarity. Please see my comments below in the order they appear in text.

1. It would be useful in the introduction to clarify the concept of chronic recurrent UTI (i.e., the difference between the concept of re-infection vs. the persistence of the bacterium in bladder epithelial cells and re-emergence due to some trigger).

2. L54-55. Please reference the following statement: “Alternative therapeutic strategies such as phage therapy (PT) and fecal microbiota transplantation (FMT) have been proposed to address this clinical challenge.”

3. L66-67. “Here we report our rationale…”
Please adjust to PT, antibiotics and FMT

4. L68-69. What do the authors mean by the phrase:
“in anticipation of providing in-depth sample analysis to support protocol development and preparation for a RCT.” I understand the idea of preliminary investigation for RCT design but what is the in-depth sample analysis referring to?

5. Supplementary information 1. Why is the age provided as a decade long range? What does “improved bowel function” following FMT refer to for patient 1? Also, please clarify if this is referring to the current FMT received in this study.

6. L84-85. The statement “Pre-treatment E. coli isolates were found to be genetically stable by whole genome sequencing (WGS)” requires some evidencing. If genomes are available, then a comparative ANI or k-mer analysis would help confirm this.

7. Supplementary table 1. This table would benefit from a more detailed caption for clarity.

8. L85-86. The authors state: “consistently susceptible to co-trimoxazole (Supplementary Table 2)”. I may have misunderstood something but how does this statement align with data from patient 3 that shows resistance (Feb 2023)?

9. Supplementary figure 1B. If the data presented in bacterial CFU, why do the authors see increases with increased phage cocktail levels?

10. L87-91. These lines report on a substantial amount of results with no data shown.

11. Could the authors provide a rationale for antibiotic conditioning with oral vancomycin prior to FMT if the purpose of FMT was E. coli displacement in the gut?

12. Figure 1. It would be useful to include in the figure that only 2/3 patients received FMT.

13. L118-119 / Supplementary figure 3. How does the statement align with the fact that patient 1 had higher bacterial counts at the end when compared to baseline?

14. Supplementary figure 4. What does one-sided comparisons mean? What is the specific statistical test used?

15. Supplementary table 3. This would benefit from a better representation of phylogeny. Phylogeny based on ANI, k-mer or the current MLST would be more visually informative than tabular format.

16. Supplementary figure 7. This is a PCoA not PCA, please adjust in text. Also, it is stated that this represents Bray-Curtis dissimilarity but in the methods the authors report using Jensen-Shannon divergence?

17. Figure 2C. This figure does not quite capture L173-178. Is there a way to visually represent all that is mentioned in those lines?

18. Do the authors have any information on the species strain richness of E. coli in the urinary tract? Can there only be one strain at a time, similar to that observed for B. fragilis in the gut?

19. There is emphasis on phage therapy in the discussion but to me patient 3 who was not on FMT had the worst outcome (Figure 2C) and the success of patient 2 seems attributable to strain replacement which the assumption is it is coming from the FMT donor(?)

20. As follow on from above, it would be important to look at the metagenome-assembled genome of E. coli from the donor FMT vs patient E. coli genome at baseline vs patient E. coli genome post intervention to determine if the donor contributed the less pathogenic strain that displaced the patient strain.

21. The illustrations, particularly those in supplementary material, would benefit from improved labelling, expanded legends, larger fonts, and so on.

Online Methods

22. What is the origin of the phages used in the study?

23. The FMT appears to have originated from two separate donors based on PCoA. Please provide more details.

24. The FMT protocol cites a paper from 2015 regarding the capsules that itself cites the seminal NEJM RCT from 2011 with regard to donor screening. Donor screening protocols have been updated quite substantially since the transfer of antibiotic-resistant E. coli through FMT. Can the authors clarify the protocols used and cite more up-to-date literature if available from the bank?

Reviewer #3 (Remarks to the Author):

TThis paper addresses the treatment of recurrent urinary tract infections (rUTIs), a debilitating condition that often leads to repeated antibiotic courses and contributes to antimicrobial resistance. The concept of combining phage therapy with faecal microbiota transplantation represents a novel therapeutic approach that, if successful, could substantially improve patients' quality of life while mitigating future antibiotic resistance.
However, the findings should be regarded as preliminary given the very limited number of participants. Future studies will require much larger sample sizes to establish clinical efficacy and overall effectiveness, particularly as the treatment appeared effective in only two of the three participants. Furthermore, the study lacks a control group against which treatment effectiveness can be compared and provides limited assessment of immune responses to treatment or long term prognosis. These issues should be explicitly acknowledged as limitations within the manuscript, together with a clearer discussion of future research needs. Overall, the study would benefit from being framed more explicitly as a proof of concept pilot investigation.
Areas for authors to address
1. UTI diagnostic criteria
This study followed three individuals with recurrent UTIs. Could the authors clarify the specific diagnostic criteria used to confirm UTI? For example, was diagnosis based on:
o patient reported symptoms alone;
o microbiological findings (e.g. specified CFU thresholds); or
o a combination of clinical and laboratory parameters?
2. Phage cocktail composition
How many distinct phage strains were included in the therapeutic cocktail, and how were they selected? This information is essential for understanding the breadth of bacterial targeting and the potential for resistance development.
3. E. coli monitoring and analysis
The monitoring of urinary E. coli titres shows a gap between day 8 and day 12. As co trimoxazole administration overlaps with this period, it is difficult to disentangle the respective contributions of phage therapy and antibiotics to the observed reduction in bacterial load. More frequent quantitative cultures during and immediately after antibiotic exposure, particularly between days 8–12, would strengthen interpretation of the treatment effects and help clarify whether the observed bacterial decline can be attributed primarily to phage therapy, co trimoxazole, or their combination.
4. FMT screening and characterisation of E. coli
The manuscript states that FMT capsules were produced from healthy donors who were screened for transmissible pathogens. However, it is unclear whether the FMT material contained E. coli and, if so, how these strains were characterised. Given that many E. coli strains are commensal, whereas only specific lineages (e.g. uropathogenic E. coli) are associated with rUTI, it would be important to clarify:
o whether E. coli was present in the FMT product; and
o whether donors were screened specifically for uropathogenic E. coli strains.
5. Patient selection criteria
Only three patients were included, yet they represent a wide age range. Individuals over the age of 65 commonly have multiple comorbidities that may contribute to rUTI, making the findings more difficult to interpret. Could the authors provide detailed participant selection criteria, including explicit inclusion and exclusion criteria (with justification), age range specifications, assessment of comorbidities, and the rationale for the observed demographic heterogeneity? This information is essential for

defining the target patient population, enabling replication of the study, and interpreting the clinical significance of the reported outcomes.

****

Version 1:

Reviewer comments:

Reviewer #1

(Remarks to the Author)
I thank the authors for their responses and for revising the manuscript accordingly.

If I'm not mistaken, the two-phage cocktail was applied empirically, with retrospective analysis showing that the cocktail matched the patients' E. coli strains. It would be good to stress this (and the reason for it) in the text.

The scientific literature suggest that one would need a cocktail of 6-10 phages to realistically target most of the E. coli strains involved in local urinary tract infections. Applying the two-phage cocktail empirically in three patients (with retrospective confirmation of phage susceptibility) suggests that the authors were either relatively lucky (or were there patients for which phage therapy was aborted due to non-susceptibility?), or confident that the two-phage cocktail would target most local strains. It is therefore a pity that no more information regarding these phages is provided. The two phages are being engineered to produce an additional antimicrobial protein and therefore no more details can be provided. The preclinical development of the cocktail will be published in a separate paper. This suggests that more patients will be treated with these two wildtype phages, indicating an exceptional host range, and this awaiting the development of the engineered cocktail.

Historically, LB is the abbreviation of "lysogeny broth" (developed in 1951 by Bertani). Luria-Bertani is a later, common, but not original interpretation.

The Supplementary Material PDF file ends with a reprint of Figures 1 and 2 and a 280-page comparison of bacterial genomes.

Reviewer #2

(Remarks to the Author)
The authors have addressed all my comments in the revised manuscript, thank you. Good luck with the clinical trial.

Reviewer #3

(Remarks to the Author)
Thank you for updating and addressing my comments.
For UTI diagnosis, did you go by CFU thresholds for the number of E. coli present in the urine sample to be considered as a UTI?

Decision Letter:

20th March 2026

Dear Shawna,

Thank you for commenting on the questions that I had sent you regarding your Brief Communication entitled "Phage therapy plus fecal microbiota transplantation to treat recurrent urinary tract infection: a case series". I have now discussed this with our chief editor, and we will need you to address the following:

Please add the following information to your manuscript:

> Please provide information on how the two-phage cocktail was selected in the results and methods. Was this an existing cocktail or a personalised treatment?

> Please provide an explanation for why FMT was used alongside phage therapy and antibiotics for these patients, rather than phage and antibiotics alone.

> Please add the proposed wording and mention this information in the results as well as the methods.

"A two-phage cocktail consisting of two wildtype E. coli phages, E2 (Accession OL870316; Du et al., 23) and phi41S, belonging to the genera Tequatrovirus and Phapecoctavirus, were used based on complementary host range and different receptors. Both

phages were isolated from environmental wastewater sources in Switzerland and were purified for these treatments using caesium chloride (CsCl)."

Please also note that we cannot allow reference to unpublished data or manuscripts in preparation, unless cited as a preprint.

Please use the link below to submit a revised paper:

Link Redacted

Nature Microbiology is committed to improving transparency in authorship. As part of our efforts in this direction, we are now requesting that all authors identified as 'corresponding author' on published papers create and link their Open Researcher and Contributor Identifier (ORCID) with their account on the Manuscript Tracking System (MTS), prior to acceptance. This applies to primary research papers only. ORCID helps the scientific community achieve unambiguous attribution of all scholarly contributions. You can create and link your ORCID from the home page of the MTS by clicking on 'Modify my Springer Nature account'. For more information please visit please visit www.springernature.com/orcid.

Could you please resubmit the revised manuscript by March 27, 2026? Thank you.

Yours sincerely,

Version 2:

Decision Letter:

Our ref: NMICROBIOL-25093401B

2nd April 2026

Dear Shawna,

Thank you for submitting your revised manuscript "Phage therapy plus fecal microbiota transplantation to treat recurrent urinary tract infection: a case series" (NMICROBIOL-25093401B). Editorially, we find that the paper has improved in revision, and therefore we'll be happy in principle to publish it in Nature Microbiology, pending minor revisions to comply with our editorial and formatting guidelines.

We are now performing detailed checks on your paper and will send you a checklist detailing our editorial and formatting requirements in about two weeks. Please do not upload the final materials and make any revisions until you receive this additional information from us.

Thank you again for your interest in Nature Microbiology. Please do not hesitate to contact me if you have any questions.

Best wishes,

Version 3:

Decision Letter:

28th May 2026

Dear Shawna,

I am pleased to accept your Brief Communication "Combined Phage therapy and fecal microbiota transplantation to treat recurrent urinary tract infection: a case series" for publication in Nature Microbiology. Thank you for having chosen to submit your work to us and many congratulations.

Over the next few weeks, your paper will be copyedited to ensure that it conforms to Nature Microbiology style. We look particularly carefully at the titles of all papers to ensure that they are relatively brief and understandable.

Once your paper is typeset, you will receive an email with a link to choose the appropriate publishing options for your paper and our Author Services team will be in touch regarding any additional information that may be required. Once your paper has been scheduled for online publication, the Nature press office will be in touch to confirm the details.

You may wish to make your media relations office aware of your accepted publication, in case they consider it appropriate to

organize some internal or external publicity. Once your paper has been scheduled you will receive an email confirming the publication details. This is normally 3-4 working days in advance of publication. If you need additional notice of the date and time of publication, please let the production team know when you receive the proof of your article to ensure there is sufficient time to coordinate. Further information on our embargo policies can be found here:
https://www.nature.com/authors/policies/embargo.html

After the grant of rights is completed, you will receive a link to your electronic proof via email with a request to make any corrections within 48 hours. If, when you receive your proof, you cannot meet this deadline, please inform us at rjsproduction@springernature.com immediately. You will not receive your proofs until the publishing agreement has been received through our system

Due to the importance of these deadlines, we ask you please us know now whether you will be difficult to contact over the next month. If this is the case, we ask you provide us with the contact information (email, phone and fax) of someone who will be able to check the proofs on your behalf, and who will be available to address any last-minute problems.

Acceptance of your manuscript is conditional on all authors' agreement with our publication policies (see https://www.nature.com/nmicrobiol/editorial-policies). In particular your manuscript must not be published elsewhere.

Authors may need to take specific actions to achieve compliance with funder and institutional open access mandates. If your research is supported by a funder that requires immediate open access (e.g. according to Plan S principles or the NIH public access policy) then you should select the gold OA route, and we will direct you to the compliant route where possible. Because authors warrant under our subscription licensing terms that they haven’t committed to licensing any version of their article under a licence inconsistent with the terms of our agreement – including the applicable embargo period – publication under the subscription model isn’t suitable for authors whose funders require no embargo.

If you have any questions about our publishing options, costs, Open Access requirements, or our legal forms, please contact ASJournals@springernature.com

An online order form for reprints of your paper is available at https://www.nature.com/reprints/author-reprints.html. All co-authors, authors' institutions and authors' funding agencies can order reprints using the form appropriate to their geographical region.

We welcome the submission of potential cover material (including a short caption of around 40 words) related to your manuscript; suggestions should be sent to Nature Microbiology as electronic files (the image should be 300 dpi at 210 x 297 mm in either TIFF or JPEG format). Please note that such pictures should be selected more for their aesthetic appeal than for their scientific content, and that colour images work better than black and white or grayscale images. Please do not try to design a cover with the Nature Microbiology logo etc., and please do not submit composites of images related to your work. I am sure you will understand that we cannot make any promise as to whether any of your suggestions might be selected for the cover of the journal.

You can now use a single sign-on for all your accounts, view the status of all your manuscript submissions and reviews, access usage statistics for your published articles and download a record of your refereeing activity for the Nature journals.

To assist our authors in disseminating their research to the broader community, our SharedIt initiative provides you with a unique shareable link that will allow anyone (with or without a subscription) to read the published article. Recipients of the link with a subscription will also be able to download and print the PDF.

As soon as your article is published, you will receive an automated email with your shareable link.

Congratulations once again and I look forward to seeing the article published.

With kind regards,

P.S. Click on the following link if you would like to recommend Nature Microbiology to your librarian
http://www.nature.com/subscriptions/recommend.html#forms

** Visit the Springer Nature Editorial and Publishing website at www.springernature.com/editorial-and-publishing-jobs for more information about our career opportunities. If you have any questions please click here.**

Open Access This Peer Review File is licensed under a Creative Commons Attribution 4.0 International License, which permits use, sharing, adaptation, distribution and reproduction in any medium or format, as long as you give appropriate credit to the original author(s) and the source, provide a link to the Creative Commons license, and indicate if changes were made.
In cases where reviewers are anonymous, credit should be given to 'Anonymous Referee' and the source.
The images or other third party material in this Peer Review File are included in the article's Creative Commons license, unless indicated otherwise in a credit line to the material. If material is not included in the article's Creative Commons license and your intended use is not permitted by statutory regulation or exceeds the permitted use, you will need to obtain permission directly from the copyright holder.
To view a copy of this license, visit https://creativecommons.org/licenses/by/4.0/

Response to Referees

Dear Referees and Editorial Team,

We would like to thank the three Reviewers and the corresponding Editor who reviewed the submission of our case series on using phage therapy plus FMT to treat recurrent UTI (NMICROBIOL-25093401A). We have now addressed your queries and have provided detailed responses to each point below, where our comments are provided in *blue italics* following each comment. The text has been shortened in multiple areas in order to adhere to the "Brief Communication" format for Nature Microbiology, and the information has been shifted to the Online Methods or Supplementary documents. Information may also have been reorganized in function of the CARE guidelines for case reports. This is the first time patients have been treated with such a combination approach. While this was done in individual cases in this setting, we have started preparation for a randomized controlled trial. We thank you again for your expertise and time that you dedicated to improving our manuscript.

For quick orientation, please find the responses to each person at the following locations:

RESPONSE TO EDITORIAL COMMENTS: 1
RESPONSE TO REVIEWERS: 2
Reviewer #1 2
Reviewer #2 6
Reviewer #3 11

Response to editorial comments:

- methods don't include any information about the patients, ethics, written informed consent. This needs to be provided

We have added a patient paragraph to the Methodology section which also includes this information. Of note, the treatment was not within a clinical trial but as individual trials (individual healing attempts) according with a product given based on the German Medicinal Act (AMG) § 13.1 and in Switzerland as an Experimental treatment for individual cases. For this, we do not require an IRB approval as long as we do inform the patient sufficiently and document the informed consent (based on German Civil Code: § 630d BGB). However, we do have IRB approval for the biosampling and accompanying research analyses for these cases, which the patients gave a separate written informed consent to. We hope the frameworks and permissions are now clearly outlined in the manuscript.

- there is no Data Availability Statement and no mention of access to shotgun metagenomics. Please provide this information

We apologize for omitting this and the information is now added as a specific section at the end of the manuscript under "Data availability" and reads "Anonymised clinical data from the three patients included in this study have been deposited in the Phagistry (www.phagistry.org). Raw bacterial and metagenomic sequencing data reported in this manuscript have been deposited in the NCBI Sequence Read Archive (SRA) under accession number PRJNA1331746. All other raw data supporting the findings of this study and custom code used for analyses are available from the corresponding author upon request."

- is this part of a trial? We need more information about patient selection, phage selection, why different treatments were chosen etc.

We apologize if there was confusion to this point, but this was a case series and not a clinical trial. Patient information has been added to the material and methods, along with the detailed patient vignettes in Supplementary Information 1. The phages were selected based on their current development for a clinical trial and therefore the information that we can provide at this time is limited. We have, however, added additional detail and respond to the Reviewer queries with more specific information.

- do you have a research protocol you can provide? IRB approval?

Please refer to the above responses.

- as this is a case series, you cannot make claims about specific outcomes like safety or efficacy. It's purely observational. You need to report in line with the CARE guidelines (the CONSORT for case reports).

Thank you for this comment and we are cognizant of the preliminary nature of these findings. We have reworded some of the language to be more observation based and have included the CARE checklist as Supplementary Information.

Response to reviewers:

Reviewer #1

(Remarks to the Author): According to the title of the present manuscript, McCallin and colleagues share their findings from the treatment of patients with recurrent urinary tract infections using phage therapy (PT) plus fecal microbiota transplantation (FMT).

In the abstract and main text, we find out that the manuscript describes three cases, two patients treated with (semi-personalized) PT + antibiotics (cotrimoxazole and vancomycin) + FMT, and one patient treated with PT + antibiotics.

The present case series thus pertains to two patients treated with PT + antibiotics + FMT.

Questions/comments:

What is meant by semi-personalized PT?

This term is used to signify that phage susceptibility testing was performed but that it was not truly personalized in the sense that phages were not selected specifically per patient. We have removed this term to avoid any misunderstanding.

What is the rationale behind this sequence of different treatments and why was one patient not treated with FMT? Is the treatment protocol based on an in vitro study, or on previous (historical) case studies or series? Why were PT and antibiotics applied sequentially (with 1 day overlap) instead of simultaneously.

We thank the Reviewer for this question. The order of phage, antibiotics, then FMT was chosen based on the following principles: phages require bacteria to replicate and therefore would have a greater effect when bacterial titers are highest (pre-antibiotic treatment); co-trimoxazole was given at the end of the phage treatment phase to eliminate any residual bacteria (low level, potentially phage resistant) and vancomycin was for the preparation of the FMT; FMT was to replenish and replace the microbiome. We are working on developing "push-pull" strategies that aim first to eliminate pathogens and then introduce new bacteria to occupy the niche.

The reason for the one patient not receiving FMT is patient preference; as these were individual healing attempts, patients ultimately could choose if they wanted to receive FMT. Patient 1 received FMT one year before, which is now more clearly stated in the text, but did not have substantial improvement afterwards, and therefore we attempted phage therapy + FMT this round of treatment.

It is stated "Unlike antibiotics, which disrupt the microbiome and provoke overgrowth of secondary pathogens, PT's narrow specificity could preserve microbial balance, potentially reducing recurrence driven by opportunistic species". Indeed, but it is also suggested (also by the authors) that PT alone cannot achieve sustainable eradication. So, in that context, the addition of FMT makes sense. Yet, potent antibiotics were also used after PT and continued for 24 months. Was the applied antibiotic therapy adapted (e.g. less intensive) compared to patients that do not receive PT or PT plus FMT?

Thank you for highlighting the potential contradiction. We would like to point out that the antibiotics were not used as a suppressive therapy– patients received antibiotics co-trimoxazole (800/160 mg) twice daily for three days with a one day overlap with phage therapy (D8-D10) and were followed for UTI recurrence and antibiotic usage for a period of 24 months after treatment. The treatment regimens are now more clearly outlined in Figure 1 to avoid any future confusion.

It is stated that "microbial concentrations in the urine were unknown at the start of treatment (SOT)". Why were they not determined? Yet, further in the text, it is stated that "Two of the three patients presented with high E. coli titers in the urine at the SOT".

Patients had to travel to our institute in order to receive phage therapy so we started the treatment empirically and independently of the infectious/colonization status. Prior to the first administration, urine was collected and measured for the concentration of bacteria, at which point it was discovered that two of the three patients had high levels of bacteriuria (without symptoms). This statement was removed from the text due to length.

Not much is mentioned regarding the used phages. On what basis were these two phages chosen? What are their host ranges, bacterial receptors, interactions, activities of each

phage against the patients' E. coli strains. In other words, is there a rationale for using these two particular phages, together?,,..

The two phages used in this case series are both obligatory lytic myoviruses. They are currently under development as a two-phage cocktail that has been additionally engineered to produce an additional antimicrobial protein in collaboration with the Martin Loessner lab at the ETHZ (Du et al., 2023). We are restricted at this time as to how much detail we can provide, but they do utilize different receptors and have complementary host ranges. We plan to publish the preclinical development of the cocktail in an upcoming manuscript.

For phage susceptibility testing in liquid culture, a multiplicity of infection (MOI) of 10 was chosen, which is quite high, especially for a cocktail of phages. Why was MOI 10 chosen?

This is the concentration that we often use for testing in the lab, and we find it a reasonable estimate for what would be encountered in the bladder. Given that the concentration of bacteria in the bladder was to the magnitude of 10^7 PFU/mL, we were treating with an MOI of approximately 10-100. A similar rationale was used for calculating the potential in vivo MOI in the published clinical trial results with LBP-01 for E. coli acute UTI by Kim et al., 2024, and we hope that the Reviewer finds this rationale acceptable.

Overall, the presentation and discussion of results is rather brief and sometimes incomplete (e.g., whole genome sequencing results).

We thank the Reviewer for highlighting this. Given the format of a Brief Communication, we are limited to the degree of detail we can provide in the text, but we have added more detailed about the WGS analysis of the Patient 2 strains, as it was one of our main findings.

Supplementary Table 2 shows that patient 3 presented with three pre-treatment isolates with different antibiotic susceptibility patterns. Was phage susceptibility tested for all three isolates? If so, can results be presented in Supplementary Figure 1? Were these three pre-treatment isolates genetically identical (were antibiotic resistance genes or mutations acquired?)?

Thank you for noticing this inconsistency. Patient 3 had pre-treatment E. coli detected in her urine and analysed by WGS on two different occasions, but the E. coli had the same resistance profile and cgMLST data. The third E. coli listed in the original spreadsheet, Supplementary Table 2, Patient 3, 2nd "Pre-treatment" strain) was never cultured from this patient and seemingly resulted from a database error. We apologize for the confusion and have modified the table accordingly.

Supplementary Table 3: a dendrogram visualizing similarity and distances between isolates would be nice.

Given the high sequence similarity between most isolates, a phylogenetic tree does not provide a lot of additional information. Instead, we now added a minimum spanning tree in Supplementary Figure 8. Please see the tree below that we opted not to include in the manuscript:

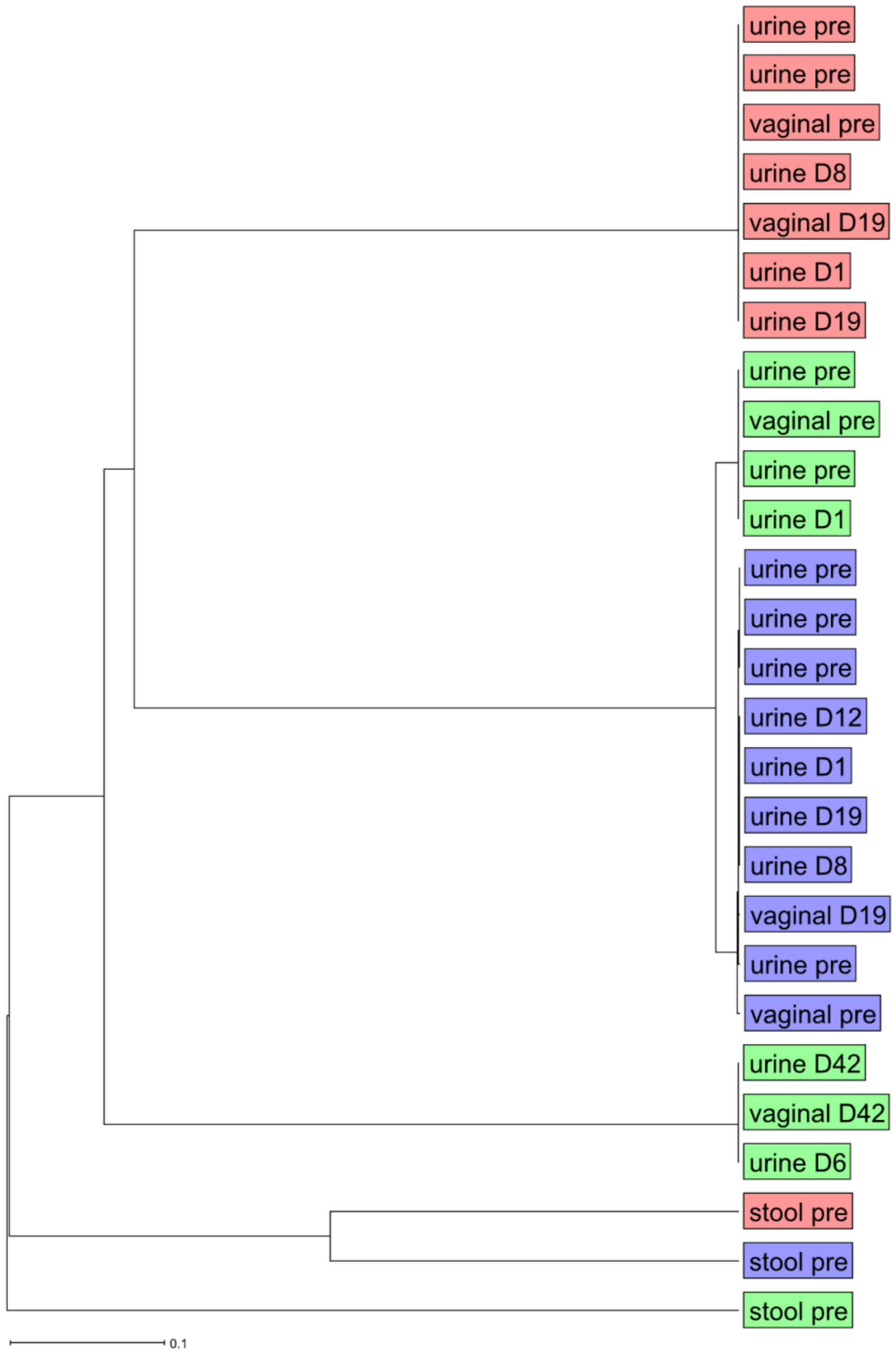

Comparison Table created: Jan 21, 2026, 10:53 AM (v11.1.2_(2026-01))

Projects: E. coli official (Escherichia coli)

E. coli cgMLST Complex Type / Clustering Distance: 10

MBioSEQ Ridom Typer UPGMA tree for 27 Samples based on 2513 columns, pairwise ignoring missing values, % columns difference

Distance based on columns from E. coli cgMLST (2513)

Patient 1

Patient 2

Patient 3

Supplementary Figure 1 could use more info, such as start concentrations, MOIs, and dilutions. This would avoid flipping back and forth to the methods section. It would also be nice to visualize the results of phage-antibiotic interaction testing.
That is a good suggestion and the mentioned information, (MOI 10, 2x10^8 PFU/mL) has been added to the legend. An additional figure, Supplementary Figure 2 now shows the results of the phage-antibiotic testing.

Reviewer #2

(Remarks to the Author): The authors have conducted a clinical study on three patients with chronic recurrent UTIs that examines the preliminary efficacy of an experimental treatment that involves oral and systemic phage administration and antibiotic intervention. Two of the three patients then received antibiotic conditioning (vancomycin) and oral encapsulated FMT. The authors report variable clinical success of the therapeutic strategy that is to some extent disconnected from the microbiological outcomes (i.e., persistence of E. coli post intervention). While the study provides interesting data that can inform and guide future phage interventions and RCTs, many areas would benefit from further details and improved clarity. Please see my comments below in the order they appear in text.

1. It would be useful in the introduction to clarify the concept of chronic recurrent UTI (i.e., the difference between the concept of re-infection vs. the persistence of the bacterium in bladder epithelial cells and re-emergence due to some trigger).
Recurrent urinary tract infection (rUTI) is defined as ≥2 symptomatic episodes within 6 months or ≥3 within 12 months (EAU/AUA/IDSA guidelines). These guidelines distinguish relapse as recurrence with the same organism shortly after therapy, suggesting incomplete eradication; from reinfection, defined as recurrence with a different organism or the same organism after documented clearance.
Although experimental and animal studies indicate that uropathogenic E. coli may persist within bladder epithelial cells or other reservoirs, the clinical relevance of these mechanisms in humans remains uncertain. Direct evidence for stable intracellular reservoirs in patients is limited, and recurrence may alternatively reflect recolonization from gastrointestinal sources. Therefore, we adhere to guideline-based clinical definitions and avoid mechanistic speculation in the introduction. Therapeutically, however, we consider strategies targeting potential intestinal or intravesical reservoirs. For phage-biological reasons, only patients with recurrent episodes caused by the same pathogen were included, whereas those with changing pathogens, potentially representing another phenotype, were not considered.

2. L54-55. Please reference the following statement: “Alternative therapeutic strategies such as phage therapy (PT) and fecal microbiota transplantation (FMT) have been proposed to address this clinical challenge.”
One additional reference has been added, specifically Zare, Vehreschild, & Wagenlehner, 2022. Additional references for phage therapy or FMT can be found in the next paragraph.

3. L66-67. “Here we report our rationale…”
Please adjust to PT, antibiotics and FMT
This sentence was removed for clarity due to another Reviewer’s comment.

4. L68-69. What do the authors mean by the phrase:
"in anticipation of providing in-depth sample analysis to support protocol development and preparation for a RCT." I understand the idea of preliminary investigation for RCT design but what is the in-depth sample analysis referring to?
This was referring to setting up the pipelines for sample preparation and analysis to be used in the upcoming RCT. However, we have removed this sentence, as stated in the previous point.

5. Supplementary information 1. Why is the age provided as a decade long range? What does "improved bowel function" following FMT refer to for patient 1? Also, please clarify if this is referring to the current FMT received in this study.
The age is provided as in decade intervals to decrease the likelihood of identification of the specific patients.
Improved bowel function refers to FMT treatments the patient received in 02/2022 and 12/2022. The indication was an intestinal dysbiosis following multiple antibiotic treatments, clinically associated with permanent loose stools and flatulence and a diagnosis of "leaky gut syndrome". Gastrointestinal symptoms improved substantially after the FMT procedures, but not in relation to UTIs. This clarification is now stated in the text in Supplementary Information1.

6. L84-85. The statement "Pre-treatment E. coli isolates were found to be genetically stable by whole genome sequencing (WGS)" requires some evidencing. If genomes are available, then a comparative ANI or k-mer analysis would help confirm this.
Thank you for this comment. We now include a minimum spanning tree that illustrates the results of the core genome MLST analysis that we performed using the WGS data of the different strains. It is now included in the Supplementary Figure 8. We do not think a comparative ANI or k-mer analysis would be more informative in this setting and hopes that the Reviewer finds the new figure sufficiently informative.

7. Supplementary table 1. This table would benefit from a more detailed caption for clarity.
A caption was added "Detailed patient vignettes detailing medical history and past treatments." to the supplementary table and additional information about the patient selection process was added to the Material and Methods, as suggested by the Editor.

8. L85-86. The authors state: "consistently susceptible to co-trimoxazole (Supplementary Table 2)". I may have misunderstood something but how does this statement align with data from patient 3 that shows resistance (Feb 2023)?
We have to apologize for this misunderstanding. In our initial submission, we mistakenly listed a third E. coli in the list for patient 3 pre-treatment with a deviating resistance pattern. This was incorrect. The E. coli detected in the urine prior to treatment was consistently the same (both on the level of phenotypic resistance testing and when looking at the WGS data). The mention of this incorrect strain has been removed from the Supplementary Table 3.

9. Supplementary figure 1B. If the data presented in bacterial CFU, why do the authors see increases with increased phage cocktail levels?
This figure is showing the amount/lysis ability of phage by spot assay so that the circles are lysis zones after spots of phage cocktail dilution are plated on a bacterial lawn. This shows rather plaque forming units (PFU) than CFU. The higher concentrations of phage cocktail to

the left show the highest degree of lysis against the strain and each consecutive spot is a 10x dilution.

10. L87-91. These lines report on a substantial amount of results with no data shown.
Thank you for highlighting an area that requires better data support. We opted not to show the phage susceptibility results as our genomic analysis revealed that the stool and urine isolates were genetically different. Phage-antibiotic interactions were tested in a checkerboard assay and the results are now present in a new supplementary figure (new Supplementary Figure 2). We removed the statement about serum neutralization at this place due to length, however this is now mentioned later in the text and is supported by a supplementary figure.

11. Could the authors provide a rationale for antibiotic conditioning with oral vancomycin prior to FMT if the purpose of FMT was E. coli displacement in the gut?
There is increasing evidence that engraftment of the FMT product is enhanced by prior antibiotic pretreatment (see PMID: 35931074). The most common antibiotic for this purpose is vancomycin, which is thought to disrupt the host's microbiome sufficiently. A brief rationale for Vancomycin is now mentioned in the manuscript in the Online Methods section, "Dosing regimen and treatment details".

12. Figure 1. It would be useful to include in the figure that only 2/3 patients received FMT.
This clarification has been added in the text and in the figure.

13. L118-119 / Supplementary figure 3. How does the statement align with the fact that patient 1 had higher bacterial counts at the end when compared to baseline?
This figure (previous Supplementary Figure 3A, now Supplementary Figure 4A,) shows the amount of total bacteria measured by urine flow cytometry and therefore can include non-target species. Indeed, we detected levels of non-target bacteria at different timepoints through the treatment phase and this could have contributed to the increase in bacteria per microliter. Figure 2A shows that no viable E. coli (measured by colorimetric plating) were detected at the end of treatment. The statement for flow cytometry data now reads "Leucocyte counts determined by urine flow cytometry decreased during phage application, whereas bacterial counts fluctuated and were non-specific to E. coli."

14. Supplementary figure 4. What does one-sided comparisons mean? What is the specific statistical test used?
Thank you for pointing this out. We clarified this in the legends now, what is not Supplementary Figure 5. By "one-sided" we mean a directional test if serum neutralization increases over time-for instance if the residual titers are lower at EOT than SOT. The specific test was a one-sided, unpaired two-sample t-test on log10-transformed residual titers.

15. Supplementary table 3. This would benefit from a better representation of phylogeny. Phylogeny based on ANI, k-mer or the current MLST would be more visually informative than tabular format.
We agree that an illustration is more informative. Supplementary Figure 8, a minimum spanning tree, has been added and the tables have been removed.

16. Supplementary figure 7. This is a PCoA not PCA, please adjust in text. Also, it is stated

that this represents Bray-Curtis dissimilarity but in the methods the authors report using Jensen-Shannon divergence?

Thank you for noticing this. We corrected the figure legend (PCoA and not PCA). The text in the methodology section was also corrected, we did not use Jensen-Shannon divergence in this case but looked at the Bray-curtis beta diversity.

17. Figure 2C. This figure does not quite capture L173-178. Is there a way to visually represent all that is mentioned in those lines?

Thank you for highlighting this - the main quantitative outcome was the number of UTI episodes that is displayed in the figure. The symptom improvement during PT is visually represented in Supplementary Figure 2 with the Acute Cystitis Symptom Score, which was only recorded during the PT phase of treatment. Other symptom improvement and antibiotic use was self report and of qualitative nature and therefore less conducive to visual display. We have added patient perspectives in the supplementary perspectives and emphasized the self-reported nature of some information.

18. Do the authors have any information on the species strain richness of E. coli in the urinary tract? Can there only be one strain at a time, similar to that observed for B. fragilis in the gut?

We thank the Reviewer for this question and the urinary microbiome is an ongoing research focus in our groups. While we have occasionally observed urine cultures with two E. coli strains, we cannot confidently say at this time how common it is or if this was the case for our patients. From our metagenomical analysis, we could detect the strain we were looking for by mapping reads to the house keeping genes of the ST type of the individually sequenced strains, but were not able to confidently analyse additional E. coli sequences. Thanks to this question, we looked again and were not able to find the ST of the replacement strain of Patient 2 (ST73) in any of the pre-treatment samples so it is still uncertain where it originated from.

19. There is emphasis on phage therapy in the discussion but to me patient 3 who was not on FMT had the worst outcome (Figure 2C) and the success of patient 2 seems attributable to strain replacement which the assumption is it is coming from the FMT donor(?)

Thank you for pointing out this imbalance. Patient 3 did benefit the least from the experimental treatment, but we cannot confidently say that FMT would have changed this outcome. Interestingly, the strain replacement that seems to have resulted in a benefit for Patient 2 occurred prior to FMT (i.e. during PT) and we were not able to locate it in the urine or stool metagenomes prior to starting PT by mapping reads to the house keeping genes of the new strain.

20. As follow on from above, it would be important to look at the metagenome-assembled genome of E. coli from the donor FMT vs patient E. coli genome at baseline vs patient E. coli genome post intervention to determine if the donor contributed the less pathogenic strain that displaced the patient strain.

The strain replacement was observed in a sample taken prior to the FMT, so we conclude that the FMT donor was not the source of the emerging strain in patient 2. However, we checked the metagenome sequences of the donor stool sample. It contained only few E. coli reads (5.8×10^{-6} %), which did not map against the MLST genes of the replacement strain of patient 2 (ST73). We specifically looked at alleles that clearly separate ST17729

(initial E. coli of patient 2) vs ST73, but the findings were limited due to the low abundance. The information on E. coli abundance in donor stool is now added to the main text.

21. The illustrations, particularly those in supplementary material, would benefit from improved labelling, expanded legends, larger fonts, and so on.
Thank you for this comment and we have revised the supplementary material accordingly.

Online Methods

22. What is the origin of the phages used in the study?
The phages were both isolated from waste water sources in Switzerland.

23. The FMT appears to have originated from two separate donors based on PCoA. Please provide more details.
Correct, the FMT products were from two different donors. We have a FMT bank (Cologne Microbiota Bank) with different donors. Selection is based on availability of a released product.

24. The FMT protocol cites a paper from 2015 regarding the capsules that itself cites the seminal NEJM RCT from 2011 with regard to donor screening. Donor screening protocols have been updated quite substantially since the transfer of antibiotic-resistant E. coli through FMT. Can the authors clarify the protocols used and cite more up-to-date literature if available from the bank?
Thank you for this comment. Unfortunately, we do not have a more recent publication explaining our donor screening strategy in detail. However, you are correct, that donor screening has evolved over the past years. Our institution has participated and is still participating in European guidance and guideline development on screening procedures in FMT and our screening follows the 5th edition of the EDQM "Guide to the quality and safety of tissue and cells for human application, 2022 . Of note, we have always screened for multidrug-resistant gram negative bacteria, also prior to the reports of the bloodstream infections by resistant E. coli.
Since the current paper already has a very comprehensive supplementary body, we decided not to include a comprehensive list of all pathogens we screen for, but added a brief explanation with regards to the E. coli screening approach in the Methodology section. For your information, please find the comprehensive list of pathogens we test for in stool in the table below. In addition we test for HIV, hepatitis and Syphilis in blood.

Table: List of pathogens screened for as part of the FMT donor selection process

Organism	Method	Acceptance criteria
Helicobacter pylori	ELISA (Antigenic)	Negative
E.g. G. lamblia, D. fragilis, Helminthen, Entamoeba spp., S. stercoralis, Isospora spp.	MIFC	No cysts, eggs, worms, worm limbs
Noroviruses	PCR	Negative

C. difficile (tcdB)	Toxin-PCR	Negative
EHEC/STEC (Ggfs. O157)	PCR	Negative
ETEC	PCR	Negative
EPEC	PCR	Negative
EIEC	PCR	Negative
EAggEC	PCR	Negative
Plesiomonas shigelloides	PCR	Negative
Campylobacter spp.	PCR	Negative
Yersinia enterolytica	PCR	Negative
Vibrio spp.	PCR	Negative
Vibrio cholerae	PCR	Negative
Salmonella spp.	PCR	Negative
Rotavirus A	PCR	Negative
Adenovirus	PCR	Negative
Astrovirus	PCR	Negative
Sapovirus	PCR	Negative
Giardia lamblia	PCR	Negative
Entamoeba histolytica	PCR	Negative
Cryptosporidium spp.	PCR	Negative
Dientamoeba fragilis	PCR	Negative
Cyclospora cayetanensis	PCR	Negative
Ancylostoma spp.	PCR	Negative
Ascaris spp.	PCR	Negative
Enterobius vermicularis	PCR	Negative
Mikrosporidien	PCR	Negative
Hymenolepis spp.	PCR	Negative
Necator americanus	PCR	Negative
Strongyloides spp.	PCR	Negative
Taenia spp.	PCR	Negative
Trichuris trichiura	PCR	Negative
MRSA	Culture	Negative
VRE	Culture	Negative
3MRGN	Culture	Negative
4MRGN	Culture	Negative
Candida auris	Culture	Negative
Listeria monocytogenes	Culture	Negative
Enteroviruses	PCR	Negative
SARS-CoV-2	PCR	Negative

Reviewer #3

(Remarks to the Author): This paper addresses the treatment of recurrent urinary tract infections (rUTIs), a debilitating condition that often leads to repeated antibiotic courses and contributes to antimicrobial resistance. The concept of combining phage therapy with faecal microbiota transplantation represents a novel therapeutic approach that, if successful, could

substantially improve patients’ quality of life while mitigating future antibiotic resistance. However, the findings should be regarded as preliminary given the very limited number of participants. Future studies will require much larger sample sizes to establish clinical efficacy and overall effectiveness, particularly as the treatment appeared effective in only two of the three participants. Furthermore, the study lacks a control group against which treatment effectiveness can be compared and provides limited assessment of immune responses to treatment or long term prognosis. These issues should be explicitly acknowledged as limitations within the manuscript, together with a clearer discussion of future research needs. Overall, the study would benefit from being framed more explicitly as a proof of concept pilot investigation.

Areas for authors to address
1. UTI diagnostic criteria
This study followed three individuals with recurrent UTIs. Could the authors clarify the specific diagnostic criteria used to confirm UTI? For example, was diagnosis based on:
o patient reported symptoms alone;
o microbiological findings (e.g. specified CFU thresholds); or
o a combination of clinical and laboratory parameters?
The selected patients all had frequent UTIs that presented with both, urinary symptoms (see Supplementary information 1) and detection of E. coli in corresponding urine samples. Patients did not have an acute episode during the course of the treatment of PT+FMT. Additional information about the patient selection process has been added in the Material and Methods section.

2. Phage cocktail composition
How many distinct phage strains were included in the therapeutic cocktail, and how were they selected? This information is essential for understanding the breadth of bacterial targeting and the potential for resistance development.
The phage cocktail was comprised of two distinct phages that were isolated from wastewater sources in Switzerland and were selected for their complementary host ranges (cocktail breadth) and use of different bacterial receptors (cocktail depth). They are currently under development as a two-phage cocktail that has been additionally engineered to produce an additional antimicrobial protein in collaboration with the Martin Loessner lab at the ETHZ (Du et al., 2023). We are restricted at this time as to how much detail we can provide, but they do utilize different receptors and have complementary host ranges. We plan to publish the preclinical development of the cocktail in an upcoming manuscript.

3. E. coli monitoring and analysis
The monitoring of urinary E. coli titres shows a gap between day 8 and day 12. As co trimoxazole administration overlaps with this period, it is difficult to disentangle the respective contributions of phage therapy and antibiotics to the observed reduction in bacterial load. More frequent quantitative cultures during and immediately after antibiotic exposure, particularly between days 8–12, would strengthen interpretation of the treatment effects and help clarify whether the observed bacterial decline can be attributed primarily to phage therapy, co trimoxazole, or their combination.
We thank the Reviewer for this comment and agree that it is not possible to separate the different treatment effects from one another. Additional sampling was not done due to feasibility that the patients traveled to Switzerland to receive phage therapy and were at

home between the PT and FMT treatments. We will consider adding additional time points to measure E. coli between this time frame in the future, although it is our experience that urine cultures are negative if the patients are receiving antibiotics.

4. FMT screening and characterisation of E. coli
The manuscript states that FMT capsules were produced from healthy donors who were screened for transmissible pathogens. However, it is unclear whether the FMT material contained E. coli and, if so, how these strains were characterised. Given that many E. coli strains are commensal, whereas only specific lineages (e.g. uropathogenic E. coli) are associated with rUTI, it would be important to clarify:
o whether E. coli was present in the FMT product; and
o whether donors were screened specifically for uropathogenic E. coli strains.
Please also see reply to reviewer 2, comment 24. We usually do not exclude donors based on presence of E. coli. We have added information on the extent of E. coli screening (referring to pathogenic but not uropathogenic E. coli and to multidrug-resistant E. coli) in the Methodology. In addition, we now looked at the presence of E. coli in the FMT products based on metagenomic sequencing: The FMT product that patient 1 received did not have any E. coli reads based on our sequencing results. The FMT product that patient 2 received had a very low abundance of 5.8 x 10^{-6} % and this information is now stated in the manuscript.

5. Patient selection criteria
Only three patients were included, yet they represent a wide age range. Individuals over the age of 65 commonly have multiple comorbidities that may contribute to rUTI, making the findings more difficult to interpret. Could the authors provide detailed participant selection criteria, including explicit inclusion and exclusion criteria (with justification), age range specifications, assessment of comorbidities, and the rationale for the observed demographic heterogeneity? This information is essential for defining the target patient population, enabling replication of the study, and interpreting the clinical significance of the reported outcomes.
These three patients were all seen in rUTI clinics in Frankfurt and Cologne and had a persistent detection of E. coli plus a broad range of unsuccessful treatment attempts. This combination served as a justification for us to conduct an individual treatment attempt with this international consortium. Of note, they were not selected out of a larger list of eligible patients but they were the first three that fulfilled these criteria. As we hope to test this concept in a clinical trial setting, we will have clear inclusion and exclusion criteria that will minimize the heterogeneity of the patient population.

Response to Editors

Dear Editorial Team,

We would like to thank you for the time and attention you have accorded to our submission of our case series on using phage therapy plus FMT to treat recurrent UTI (NMICROBIOL-25093401A). We have addressed the remaining queries and have provided detailed responses to each point below, where our comments are provided in *blue italics* following each comment. We thank you again for your expertise and time that you dedicated to improving our manuscript.

> Please provide information on how the two-phage cocktail was selected in the results and methods. Was this an existing cocktail or a personalised treatment?

The word "existing" was added on line 66 to specify that this cocktail was pre-existing. While phages were not chosen per patient, it is still personalized to the extent that phage susceptibility guided the decision to treat.

> Please provide an explanation for why FMT was used alongside phage therapy and antibiotics for these patients, rather than phage and antibiotics alone.

The rationale for using FMT to restore the intestinal microbiome with the intention to prevent future UTI recurrence has been added on line 52-53 and 181-183, where we state "FMT was intended here to reduce the likelihood of recurrence of UTI due to a more protective microbiome; something that PT alone does not provide."

> Please add the proposed wording and mention this information in the results as well as the methods.

"A two-phage cocktail consisting of two wildtype E. coli phages, E2 (Accession OL870316; Du et al., 23) and phi41S, belonging to the genera Tequatrovirus and Phapecoctavirus, were used based on complementary host range and different receptors. Both phages were isolated from environmental wastewater sources in Switzerland and were purified for these treatments using caesium chloride (CsCl)."

This was added to the main text on lines 67-71 and again in the online methods, lines 306-310. We added one additional statement about their application at bedside on line 270 "Phages were stored separately and mixed at bedside prior to administration". The phage names, E2 and phi41S, have been specified in Supplementary Figures 5 and 7, as well in the certificate of analysis reports for the phage quality control. Two co-authors have been added who we have collaborated with on the cocktail creation.

Please also note that we cannot allow reference to unpublished data or manuscripts in preparation, unless cited as a preprint.

This has been removed.
